# Experimental Validation of Genome‐Environment Associations in Arabidopsis

**DOI:** 10.1111/mec.70129

**Published:** 2025-10-06

**Authors:** Yuxin Luo, Claire M. Lorts, Erica H. Lawrence‐Paul, Jesse R. Lasky

**Affiliations:** ^1^ Department of Biology Pennsylvania State University University Park Pennsylvania USA; ^2^ PAC Herbarium Pennsylvania State University University Park Pennsylvania USA

**Keywords:** genome‐environment associations (GEA), local adaptation, *LSD1*, t‐DNA knockout, *WRKY38* drought stress

## Abstract

Identifying the genetic basis of local adaptation is a key goal in evolutionary biology. Allele frequency clines along environmental gradients, known as genotype‐environment associations (GEA), are often used to detect potential loci causing local adaptation but are rarely followed by experimental validation. Here, we tested loci identified in three moisture‐related GEA studies on *Arabidopsis*. We studied 42 GEA‐identified genes using t‐DNA knockout lines under drought and tested effects on flowering time, an adaptive trait, and genotype‐by‐environment (GxE) interactions for performance and fitness. In total, 16/42 genes had significant effects on traits involved in local adaptation or performance responses to the environment. We found that *wrky38* mutants had significant GxE effects for fitness; *lsd1* plants had a significant GxE effect for flowering time, and 11 genes showed flowering time effects with no drought interaction. However, most GEA candidates did not exhibit GxE. In the follow‐up experiments, *wrky38* caused decreased stomatal conductance and specific leaf area under drought, indicating potentially adaptive drought avoidance. Additionally, GEA identified natural putative LoF variants of *WRKY38* associated with dry environments, as well as alleles associated with variation in *LSD1* expression. While only a few GEA‐identified genes were validated for GxE interactions for fitness, we likely overlooked some genes because experiments might not well represent natural environments and t‐DNA insertions might not well represent natural alleles. Nevertheless, GEAs apparently identified some genes contributing to local adaptation. GEA and follow‐up experiments are straightforward to implement in model systems and demonstrate prospects for GEA discovery of new local adaptations.

## 1 Introduction

A substantial portion of genetic variation within species in ecologically important traits is likely due to local adaptation to the environment (Savolainen et al. 2013; Tigano and Friesen 2016; Wadgymar et al. 2022). Local adaptation is defined by a genotype‐by‐environment (GxE) interaction where local genotypes have higher fitness than foreign genotypes (Kawecki and Ebert 2004). Identifying locally adaptive alleles and traits is of great interest in plant and animal breeding and conservation, where these alleles could be deployed for genetic improvement and prediction (Cortés et al. 2022; Crossa et al. 2017; Lasky et al. 2023; Whiting et al. 2024). Central questions of local adaptation include the effect size and number of involved mutations (Yeaman and Whitlock 2011), whether locally adapted mutations have tradeoffs among different environments (Lee et al. 2024), whether the same or different mutations are involved in local adaptation to the same environments in different populations (Ralph and Coop 2015), and which environmental gradients drive locally adaptive genetic variation (Lasky et al. 2012).

Conducting multiple common garden experiments to map populations and genetic loci contributing to fitness tradeoffs has been a gold standard approach (Clausen et al. 1941; Savolainen et al. 2013). However, such experiments are logistically challenging or impossible in many systems. Alternative approaches using geographic patterns in allele frequency have emerged for identifying loci potentially underlying local adaptation. Loci showing substantial allele frequency differences between populations, which reflect underlying environmental variables, may indicate those loci driving local adaptation (Coop et al. 2010; Endler 1973). With the increasing availability of high‐throughput genomic data, Genotype‐Environment Association (GEA) studies have been widely employed to identify putative locally adapted loci (Lasky et al. 2023; Rellstab et al. 2015).

GEAs employ diverse methods to identify loci where allele frequency is closely associated with environmental conditions (Lasky et al. 2023; Rellstab et al. 2015). Methods differ depending on whether they are single‐locus or multi‐loci (e.g., genome‐wide) models (Betancourt et al. 2021; Gehan et al. 2015; Hager et al. 2022; Hancock et al. 2011; Lasky et al. 2012; Lee et al. 2024), where the latter may capture groups of loci covarying with the environment that are potentially relevant for polygenic adaptation (Forester et al. 2018). Additionally, there are methods that control for genome‐wide patterns of similarity among populations using random effects (Zoubarev et al. 2012). While these approaches may reduce spurious associations due to population structure–environmental covariation, they can also be too conservative (Alonso‐Blanco et al. 2016). Thirdly, some approaches synthesise the evidence from GEA with additional evidence, such as multiple common garden experiments (Capblancq et al. 2023; Lasky et al. 2018; Soudi et al. 2023). While these methods are now standard approaches in ecological genomics, the findings are rarely followed by experimental validation of variation at individual loci (Monroe et al. 2018; Nyine et al. 2025; Tergemina et al. 2025).

To experimentally test the hypothesis that variation at a given locus causes GxE for fitness, approaches that isolate experimental genetic variation to that locus are the most powerful. Creating near‐isogenic lines (NILs) segregating at a given locus is ideal for testing GxE effects of natural allelic variation, but generating NILs is a time‐consuming process. Gene knockout mutants in model species like *Arabidopsis thaliana* (hereafter Arabidopsis) provide an alternative tool for testing GxE effects of variation at a particular gene. For example, many Arabidopsis knockout lines carrying t‐DNA insertions within genes that disrupt their function provide valuable resources for studying the effects of individual genes on fitness and other ecologically important traits. Although these mutants may not fully represent natural allelic variation for local adaptation, using them may be a good option for bulk experimental screening of candidate loci (Chong and Stinchcombe 2019; Monroe et al. 2018). Resequencing of large numbers of natural Arabidopsis genotypes has yielded more detailed information on functional variation (Alonso‐Blanco et al. 2016), which can be linked to loci of interest to make inferences about functional variants generating strong signals in GEA studies.

*Arabidopsis thaliana* is a small annual plant native to a wide range across Eurasia and Africa in diverse climates (Yim et al. 2024). Several studies have demonstrated evidence of adaptation along moisture gradients in *A. thaliana*, using patterns in quantitative traits (Dittberner et al. 2018), SNPs or genes (El‐Soda et al. 2015; Exposito‐Alonso et al. 2018; Hancock et al. 2011), and gene expression (Lasky et al. 2014). *A. thaliana* employs diverse strategies for drought adaptation such as drought escape via phenology and drought avoidance via traits reducing water loss (Lovell et al. 2013).

Here we focus on studying loci identified using 3 different approaches, to test whether identified genes indeed cause genotype–drought interaction effects on fitness or other aspects of performance. We are also interested in whether the genes identified as drought‐adapted in our experiments have allele frequency clines across the worldwide distribution of *A. thaliana* . For mutants identified as showing interesting genotype–environment interactions in our initial drought screen experiment, we followed up by testing their response to an additional, different drought treatment.

We tested the following hypotheses:

1. Some genes identified through GEA as potentially drought‐adapted exhibit natural variation in putative gene function based on resequencing data. Such genes would be especially amenable to using t‐DNA knockout lines for testing the effects of natural variation. Mutations in genes without natural loss‐of‐function variation can still be assessed using t‐DNA lines, though their relevance to natural variants is less clear.
2. Knockout mutants of these genes display significant genotype–drought interactions affecting traits (e.g., flowering time) known to be under changing selection from well‐watered to drought environments, fitness, or performance traits in controlled drought experiments; and
3. These genes show climate‐associated allelic variation and sequence signatures of selection, patterns consistent with adaptation to dry climates.

In our main drought screen, we found that *WRKY38* had significant GxE effects on several fitness‐related traits, and *LSD1* had a significant GxE effect on flowering time. Both *WRKY38* and *LSD1* were top candidates associated with moisture in their original studies (Lasky et al. 2018, 2012). Additionally, *WRKY38* was also found most strongly associated with winter low temperatures in a study combining GEA with data from multiple common gardens (Lasky et al. 2018), while knockouts of *LSD1* were found to affect chilling sensitivity (Huang et al. 2010), and allelic variation was associated with climate (moisture‐related, Lee et al. 2017) in Arabidopsis. Both genes happen to play a role in the salicylic acid (SA) pathway (Bernacki et al. 2019; Kim et al. 2008; Szechyńska‐Hebda et al. 2016; Wituszynska et al. 2013), primarily known for microbial defence response but which can be activated by abiotic stress (reviewed in Miura and Tada 2014; Wu et al. 2019). In light of previous studies and our results, we decided to use mutants of these genes to follow up with intermediate drought and freezing experiments, testing their possible role in adaptation to different drought and cold regimes.

## 2 Methods

### 2.1 Selecting Genes From Published Genome‐Environment Associations

We selected genes to test using five SNP lists from three published GEA studies that employed three different approaches. The first approach was based on mixed models of genome‐wide associations with climate, where we included random effects to account for genomic similarity (Kang et al. 2008). For these mixed models, we calculated SNP associations with intra‐annual monthly precipitation variability, growing season precipitation variability, or inter‐annual growing season precipitation variability (i.e., 3 separate environmental GWAS, Lasky et al. 2014). We took the SNPs from all three climate associations and combined them into one list ranked by *p*‐value for the mixed model association test. The second approach was a multivariate ordination where we identified SNPs most strongly associated with the first axis, which was associated with seasonality of temperature and precipitation, or the first axis after partialing out spatial variables, which was strongly associated with summer moisture (Lasky et al. 2012). We took SNPs with the strongest absolute loadings on each of these axes. The third approach was based on integrated information from four common gardens across Europe and also the broader ecotype panel to identify SNPs that showed the strongest SNP‐climate associations and GxE for fitness favouring home climate alleles in monthly growing season precipitation variability or aridity index (i.e., annual precipitation/PET; Lasky et al. 2018). In total, we generated 1, 2, and 2 lists of SNPs from the three studies, respectively.

From the top SNPs in each list, we only kept SNPs at least 250 kb apart. Starting from the SNP with the lowest *p*‐values, we took up to 3 closest genes within 5 kb of the SNP until we had at least 15 genes for each of the four lists of SNPs, or in the case of the combined climate association study, until we had at least 25 genes.

This procedure resulted in a list of 90 candidate genes. We chose 44 SALK lines representing knockouts of 42 genes, including 42 lines with insertions within exons and 2 lines with insertions within introns, and confirmed homozygosity using tDNA Express: Arabidopsis Gene Mapping Tool (Alonso et al. 2003; Table S1). Among the 44 SALK lines targeting 42 genes, three lines (SALK_048316C, SALK_085852C, and SALK_063969) targeting AT5G65080 (*MAF5*) and AT3G45860 (*CRK4*), although not top candidates, were included in the experiment because of their potential functional significance in drought adaptation (Caicedo et al. 2009; Ratcliffe et al. 2003; Zhao et al. 2023). As a result, 40 top‐candidate genes were validated in this study.

### 2.2 Functional Variation in the Screened Loci in the 1001 Genomes

We analysed putative functional variation in the 1001 Genomes resequencing data for all genes screened. We used the 1001 Genomes Polymorphism Browser (https://tools.1001genomes.org/polymorph/) to identify potential large‐effect mutations in each gene, which were considered as putative loss‐of‐function (LoF) variants. If more than one isoform existed for a gene, we recorded the case of the most common isoform; except when no putative LoF variant was found for this isoform, we instead used the most common isoform that had functional variants. We summarised the counts of each type of functional variation and the positions where the variations occurred.

### 2.3 Main Drought Screen

#### 2.3.1 Experimental Design and Growth Conditions

To mimic more natural growing conditions, we programmed the temperature and photoperiod in the chamber based on those of the *Lip‐0* ecotype that originated from southern Poland, representing a common field environment, following Lorts and Lasky (2020) (Table S2). Each tray represented either a drought or well‐watered treatment, and seven replicates per mutant were randomly assigned positions for each treatment. Seeds were stratified in DI water for 4–5 days before planting.

All pots were well‐watered by maintaining 2.5–5 cm of water at the bottom of each tray before drought treatments. Drought treatments began 19 days after planting when all seedlings had at least the first true leaves expanded. We removed the bottom water in the drought trays while maintaining a constant 2.5 cm of water in the well‐watered trays. To prevent the topsoil from drying, well‐watered pots were top‐watered every other day with 2.5 mL of water. The soil in drought treatment plants was allowed to dry for five weeks after the bottom water was removed; then we top‐watered each pot in both well‐watered and drought treatments with 12 mL 2× strength MIRACLE GROW solution every other day for three treatments total to maintain adequate nutrition. Trays were rotated and moved to a different bench in the growth chamber every other week. Percent volumetric water content was monitored every 30 min throughout the experiment using 3 drought‐treatment pots and 2 well‐watered pots containing *Columbia* (*Col*) and 5TE probes (METER Group Inc., Pullman, WA, USA).

#### 2.3.2 Plant Harvest and Phenotyping at Maturity

The fecundity of Arabidopsis plants that survived to reproduce was measured when all siliques reached maturity (dried and brown) and the rosette leaves had senesced. For each Arabidopsis plant, we measured inflorescence height and the number of secondary inflorescence branches (inflorescence branches arising from the inflorescence main axis). We also measured the total silique number and silique length of six siliques corresponding to the 10th, 20th, 40th, 60th, 80th, and 90th percentiles of silique positions along the inflorescence to represent silique length across the entire inflorescence (e.g., the 10th percentile silique was higher up the inflorescence than 10% of all siliques). After siliques were measured, we dried the mature inflorescence and rosette at 60°C for 24 h, weighed them separately, and then added their weights to get aboveground dry biomass. Finally, the rosette tissue was ground and used for δ^13^C and δ^15^N isotope analysis at the UC Davis Stable Isotope Facility.

### 2.4 Follow‐Up Drought and Freezing Experiments

#### 2.4.1 Intermediate Drought Experiment on *wrky38* and *lsd1*

For our intermediate drought (ID) experiment, plants were grown at 20°C/14°C (12 h/12 h) with varying watering frequencies (every 3 days for well‐watered, every 6 days for drought) starting from Day 25. At this time point, most plants had 10 leaves, which corresponds to the adult vegetative phase in *Col* (Lawrence‐Paul et al. 2023). Plants at this phase show greater genetic variation in drought response than at the earlier juvenile vegetative phase (Lawrence‐Paul et al. 2025). We grew 12 replicates of *Col*, *wrky38*, and *lsd1* each for WW and ID treatments. On Day 40, we randomly picked 4 pots per genotype per treatment for destructive measurements. Rosettes were first cut and weighed for fresh weight, and then each of them was put into a petri dish with distilled water under 4°C in the dark overnight to obtain their turgid weight. Next, all fully expanded leaves were cut, scanned, and then dried separately with the rest of the rosette tissue in an oven at 60°C for 2 weeks before leaf and rosette dry weights were both weighed. The total leaf area of each plant was analysed by ImageJ, and the Specific Leaf Area (SLA) of each plant was calculated by (Total leaf area/Total leaf dry weight). Relative Water Content (RWC) was calculated by (Rosette fresh weight−Rosette dry weight)/(Rosette turgid weight−Rosette dry weight).

Shortly before the first plant flowered, we tracked stomatal conductance (g_sw_) and Fv/fm for 10 continuous days using a Li‐600 porometer and fluorometer. Measurements were taken approximately one hour before lights were turned off. On every measurement, 4 pots of each genotype from each treatment were randomly picked, with measurements conducted on the newest fully expanded leaves that were large enough to apply the device.

Finally, we measured the same growth and fitness traits and isotopes as in the main drought screen.

#### 2.4.2 Overnight Freezing Experiment on *wrky38* and *lsd1*

To mimic the natural freezing stress that Arabidopsis could undergo in its growing season, we set the night (12 h) temperature in the growth chamber to −2°C and the day temperature to 10°C as our freezing treatment, which started on Day 30 when plants were established and had been under cold acclimation at 10°C/4°C (12/12 h) for 2 weeks. We surrounded all pots with extra soil for insulation to mimic the circumstances in nature, preventing unnaturally cold soil temperatures.

On Day 60, we measured diameter as an estimation of growth under freezing. Starting from Day 64, we shifted the temperature back to 20°C/14°C until the end of the experiment to allow plants to flower and then recorded flowering time. We grew the plants until senescence, after which we measured aboveground biomass, silique numbers, inflorescence length, and average and total silique lengths as described above. We grew 12 replicates for each genotype, and all of them survived until the end of the experiment.

### 2.5 Statistical Analyses

In both the drought experiments, we were primarily interested in whether the knockouts had genotype‐specific drought responses, i.e., whether there were genotype‐by‐drought interactions, as well as whether the knockouts altered average phenotypes under drought, i.e., whether genotype effects were significant. Thus, we applied linear mixed models (LMMs) to test traits we measured between *Col* and each mutant separately, considering genotypes as fixed effects and trays as random effects. To account for false positives arising from multiple testing, we implemented the Benjamini–Yekutieli procedure (Benjamini and Yekutieli 2001) to adjust *p*‐values using a False Discovery Rate (FDR) threshold of 0.05 (*p.adjust()*, R base). For comparison, we performed an alternative analysis using a full LMM including all lines, followed by Dunnett's Test comparing each mutant and *Col* using *emmeans()* and *contrast()* functions in package *emmeans* (Lenth 2025), which showed similar results (Tables S7–S9). We summarised how genes, originally identified in previous studies, were selected for knockout experiments and examined whether they exhibited significant genotype effects. Additionally, we calculated the ratio of genes tested with significant genotype effects relative to the total number of genes selected for screening in each original study. We also applied LMMs on the daily g_sw_ and Fv/fm data measured in the ID experiment to detect if the mutants differed from *Col* in ecophysiology on any of the 10 measuring days.

It was not feasible to control moisture in the freezing experiment as we did in the drought follow‐up because vapour pressure deficit is low at low temperatures, so we compared trait differences between each mutant and *Col* using the *DunnettTest()* function in the R package *DescTools* (Signorell et al. 2023).

### 2.6 Population Genetic Variation at *WRKY38*

Because we found some putative LoF for *WRKY38* were relatively common in the 1001 Genomes accessions, we further analysed the geographic and genetic structure of these variants. We used the imputed SNP matrix (hdf5 file) and short indels (vcf file) downloaded from the 1001 Genome Project website (https://1001genomes.org/accessions.html) for the following analyses.

We calculated Tajima's D across chromosome 5 (Chr 5) in 5‐kb windows using VCFtools (−TajimaD 5000) to test possible selection signals on *WRKY38* in natural accessions. The most common functional variant for *WRKY38* is a frameshift at position 7,495,793 on Chr 5 (Frameshift_7495793_; Table S3). To detect linkage disequilibrium and potential haplotype structure (i.e., non‐random associations among SNPs within the genomic region) between Frameshift_7495793_ and adjacent SNPs, we calculated the square of the Pearson's correlation coefficient (*r* ^*2*^). This calculation was performed between the presence/absence of Frameshift_7495793_ and the alleles of each SNP within 5 kb upstream and downstream of *WRKY38* using the *cor()* function in base R.

To characterise the geographic and population structure of natural putative LoF mutations in 1001 Genomes accessions, we constructed neighbour‐joining (NJ) trees built with the entire gene sequence of *WRKY38* (including coding and non‐coding sequences), and a genome‐wide NJ tree. We referred to the population structure of 1001 Genomes accessions in Alonso‐Blanco et al. (2016), where the authors classified 9 genetic clusters and one admixed group. For simplicity of data presentation, we combined the 9 groups by 5 major regions—West Mediterranean (W‐Med), North Mediterranean (N‐Med), Central and Western Europe (CW‐Euro), North Europe (N‐Euro), and Asia. Accessions that were introduced, classified in the admixed group, or might be potential contaminants (Pisupati et al. 2017) were not included in the 5 groups.

To build the genome‐wide tree, we annotated the vcf file using SnpEff (Cingolani et al. 2012), extracted the synonymous sites, filtered out sites with minor allele frequency below 0.01 (–maf 0.01) and missing rate over 10% (–max‐missing 0.9), and generated a distance matrix using PLINK (Purcell et al. 2007). Both NJ trees were constructed using the *nj()* function from package *ape* (Paradis and Schliep 2019).

We hypothesised that the natural *WRKY38* putative LoF variants could underlie local adaptation. To identify which climate variables might best explain *WRKY38* functional variation and be most closely tied to mechanisms of selection, we used LMMs to scan for correlations across the 19 bioclimatic variables. Worldclim 2 bioclimatic data were used (Fick and Hijmans 2017). Models were implemented using *coxme* and *kinship2* packages to include kinship in the models as random effects (Sinnwell et al. 2014; Therneau and Therneau 2015). We focused on testing for evidence of local adaptation based on variation between the following alleles: intact *WRKY38*, Frameshift_7495793_ (the most common and widespread putative LoF variant), and Frameshift_7495793&94_ (this double frameshift restored the normal open reading frame (ORF) and thus we suspected this allele to be functional). Since mixed models have low power when causal variants are correlated with the genomic background, we also used Welch's t‐test (*t.test()*) in R to compare the differences in bioclimatic variables for accessions with/out the intact gene or the putative LoF variants.

### 2.7 Variation in *LSD1* Expression

Only 5 of the total 1135 accessions had *LSD1* putative LoF mutations. Thus, we suspected that LoF may not be the major cause of the strong association between climate and the *LSD1*‐targeting SNP in Lasky et al. (2018), and adaptation at *LSD1* might be due to amino acid changes or *cis*‐regulatory variants impacting expression. Therefore, we scanned across 19 bioclimatic variables and SNPs (ref/alt alleles) within 10 kb around *LSD1* using univariate GEAs with GEMMA (Zoubarev et al. 2012) for 933 native accessions with known coordinates that were not potential contaminants (Pisupati et al. 2017). We found elevated correlations with climate at SNPs 1 kb upstream of *LSD1* and the start of the gene sequence, suggesting that the adaptation may occur at the promoter region. SNPs in these regions may influence gene expression by altering transcription factor binding motifs (Shastry 2009; Wang et al. 2005).

Thus, we further asked if these SNPs impact *LSD1* expression using the published 1001 Genomes transcriptome data (http://signal.salk.edu/1001.php; Kawakatsu et al. 2016). Among 728 accessions with published transcriptome data, 1 accession was excluded from the following analysis according to Pisupati et al. (2017). We compared the expression levels of *LSD1* between the two alleles of the top climate‐associated SNPs in the putative promoter region by Welch's t‐test (*t.test()*) in R. We also used linear models (*lm()*) to find possible associations between *LSD1* expression and bioclimatic variables. Additionally, we investigated whether SNPs with elevated correlations to climate are enriched in bioclimatic variables associated with *LSD1* expression.

## 3 Results

### 3.1 Widespread Putative Functional Variation in the Experimentally Screened Genes in the 1001 Genomes Accessions

Across all 1135 accessions in the 1001 Genomes, 1070 exhibited at least one high‐impact functional variant (i.e., putative LoF) over the 42 experimentally screened genes. Specifically, 1973 putative frameshifts occurred in 943 genotypes as well as 1116 stop‐gained mutations in 842 genotypes, appearing to be the most prevalent types of putative high‐impact variants over the identified genes (Table S3). Splice donor variants were found in 303 accessions, and splice‐acceptor, stop‐lost, and start‐lost variants were found in 29, 5, and 4 accessions, respectively.

Putative frameshifts were most common in AT1G19410 (*FBD*), occurring in 639 accessions. Frameshift variants were also common in AT5G22570 (*WRKY38*), detected in 250 accessions. Stop‐gained had the most counts in AT5G22900 (*CHX3*), with 492 accessions having the putative LoF variants. Splice‐donor was also the most prevalent in the *FBD* gene, exhibited by 220 accessions. The rest of the high‐impact effects were rare among the screened loci, suggesting some purifying selection on amino acid sequences. We found 14 accessions with splice‐acceptor variants, 2 accessions with stop‐lost, and 2 accessions with start‐lost at AT5G10960 (*CAF1l*), AT3G45860 (*CRK4*), and AT5G22570 (*WRKY38*), respectively.

Some mutations in the screened genes had potential dual effects, and such dual effects were locus‐specific. A 1‐bp deletion or insertion at the same position in AT1G76090 (*SMT3*) caused both frameshift and start‐loss, resulting in the most common dual effects observed in 95 accessions. Two insertions in AT3G45860 (*CRK4*) could each lead to dual effects of frameshift and stop‐gain, together occurring in 21 accessions. In rare cases, a 1‐bp deletion in AT5G40830 (*ICA*) caused frameshift and stop‐loss in 4 accessions, while a 3‐bp deletion in AT5G65050 (*AGL31*/*MAF2*) caused stop‐loss and disruptive‐inframe‐deletion effects in 1 accession.

### 3.2 Prevalent Treatment Effects, Variable Genotype Effects, and Few GxE Effects on Fitness and Phenology in the Main Drought Screen

**FIGURE 1:**
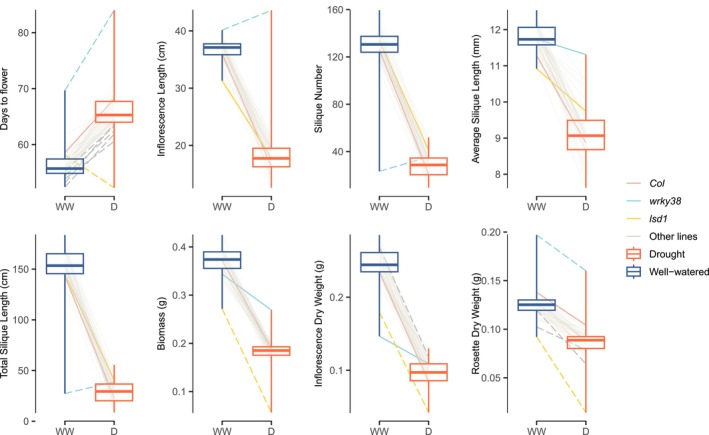
Reaction norms (lines) and average trait values (boxes) of a subset of traits measured during the main drought screen across all 44 mutants and *Col*, with *Col*, *lsd1* (CS68738), and *wrky38* (CS864818) highlighted. Each line connects the average trait values of one genotype under well‐watered (WW) and drought (D) treatments, with *Col*, *lsd1* (CS68738), and *wrky38* (CS864818) highlighted. Mutants showing significant genotype effects at FDR = 0.05 from linear mixed models (LMMs) comparing *Col* and one mutant are indicated by dashed lines.

Drought treatment effects on phenotypes were strong and largely consistent across different t‐DNA insertion lines (Figure 1). Specifically, the drought effects were significant with FDR = 0.05 across all 44 tested lines, causing lower inflorescence length and aboveground biomass, smaller inflorescence length and weight, fewer siliques, shorter average and total silique length, and delayed flowering, with line CS68738 (*lsd1*) being the only exception with no significant drought effect on flowering time (Figure 1). The drought effect on rosette weight was also common, significantly reducing rosette weights in 34 of 44 lines at FDR = 0.05.

Genotype effects varied across the 44 lines tested (Figure 1, Tables S4–S6). Genotype effects were most common for flowering time, with 22 having nominally significant effects (*α* = 0.05) on flowering, primarily accelerating flowering, except for *wrky38*, which exhibited delayed flowering under drought. Seven insertion lines were significant with FDR = 0.05 (6 accelerated flowering and 1 delayed flowering (*wrky38*)). Rosette and inflorescence weights were the traits with the next most insertion lines with genotype effects, with 18 and 12 lines having nominally significant effects and 4 and 2 significant at FDR = 0.05, which mostly led to increased rosette weight and reduced inflorescence weight. Genotype effects on other traits measured were less common, with 5, 5, 6, 5, 1 lines having nominally significant effects and 1, 0, 1, 1, 1 lines significant with FDR = 0.05 for silique number, average and total silique length, inflorescence length, and aboveground biomass, respectively.

**FIGURE 2:**
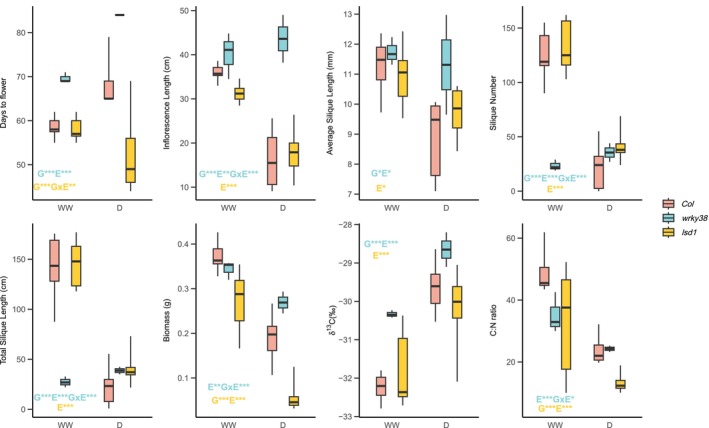
A subset of the traits measured during the main drought screen of *Col*, *lsd1* (CS68738), and *wrky38* (CS864818). Text annotations on the box plots indicate the significance of genotype (G), treatment (E), and genotype‐treatment interaction (G × E) effects at *α* = 0.05, based on LMMs comparing *Col* and one mutant. The text colour denotes the mutant genotype. Significance levels are represented as follows: *, 0.01 ≤ *p* < 0.05; **, 0.001 ≤ *p* < 0.01; ***, *p* < 0.001.

We found a few genotype‐by‐treatment interactions caused by the t‐DNA insertions. CS864818, a *WRKY38* (AT5G22570) knockout line, was the only one having a GxE effect that was significant at FDR = 0.05 on inflorescence length, silique numbers, total silique length, aboveground biomass, and inflorescence weight (Table 1, Tables S5, S6). Generally, *wrky38* showed less drought sensitivity than *Col* while being smaller and less fecund in well‐watered conditions, except that the mutant had slightly longer inflorescence lengths than *Col* (*wrky38*: 40.1 ± 3.010 cm; *Col*: 36.0 ± 0.713 cm; Figure 2). When considering only genotype effects, the knockout of *WRKY38* caused significantly later flowering, longer inflorescence, more siliques, and greater rosette weights (Figure 2, Table 1, Tables S4–S6). However, under drought conditions, only flowering time and inflorescence length were significantly different from *Col*.

**TABLE 1:** Summary statistics from mixed linear models (LMMs) assessing a subset of performance and fitness traits for *Col*, CS864818 (wrky38), and CS68738 (*lsd1*) under well‐watered and drought conditions during the main drought screen. Each LMM independently analyses *Col* and one mutant (either *wrky38* or *lsd1*). *β* represents the estimated effect size, and subscripts indicate effects attributed to genotype (*G*), treatment (Trt), or genotype‐by‐treatment interaction (GxTrt); *p* represents the *p*‐value at *α* = 0.05, and *p*_adj_ represents the adjusted *p*‐value at FDR = 0.05. A negative *β*_*G*_ reflects a lower trait value in the mutant compared to *Col*, while a positive *β*_Trt_ reflects a lower trait value under drought compared to well‐watered conditions.

*Col*‐CS864818 (*wkry38*)	Genotype effect			Treatment effect			Genotype‐by‐treatment effect		
	*β*_*G*_	*p*	*p*_*adj*_	*β*_Trt_	*p*	*p*_*adj*_	*β*_GxTrt_	*p*	*p*_*adj*_
Days to flower (days)	15.86	**< 0.0001**	**< 0.0001**	−9.57	**< 0.0001**	**< 0.0001**	−4.76	0.2191	1.0000
Inflorescence length (cm)	27.33	**< 0.0001**	**0.0014**	19.74	**< 0.0001**	**0.0086**	−23.21	**< 0.0001**	**0.0032**
Total silique length (cm)	15.82	**< 0.0001**	**0.0004**	120.03	**< 0.0001**	**< 0.0001**	−131.47	**< 0.0001**	**< 0.0001**
Biomass (g)	0.08	0.5862	1.0000	0.18	**< 0.0001**	**< 0.0001**	−0.14	**< 0.0001**	**0.0001**

*Col*‐CS68738 (*lsd1*)	Genotype effect			Treatment effect			Genotype‐by‐treatment effect		
	*β*_*G*_	*p*	*p*_*adj*_	*β*_Trt_	*p*	*p*_*adj*_	*β*_GxTrt_	*p*	*p*_*adj*_
Days to flower (days)	−15.86	**0.0002**	**0.0062**	−9.57	0.3896	1.0000	15.43	**0.0004**	0.0675
Inflorescence length (cm)	19.74	0.3868	1.0000	−6.20	**< 0.0001**	**< 0.0001**	20.14	0.0770	1.0000
Total Silique length (cm)	17.75	0.2987	1.0000	120.03	**< 0.0001**	**< 0.0001**	−15.68	0.3961	1.0000
Biomass (g)	−0.13	**< 0.0001**	**< 0.0001**	0.18	**< 0.0001**	**< 0.0001**	0.03	0.3975	1.0000

*Note:* *p*‐values less than 0.05 are presented in bold to indicate statistical significance.

Another mutant, *lsd1*, was one of only two having nominally significant genotype‐by‐treatment interaction (GxTrt) on flowering time (*p* = 0.0004; the other line SALK_012432 had *p* = 0.0370) and the only one having a trend toward significance at FDR = 0.05 (*p* = 0.0678). The mutation significantly accelerated flowering compared to *Col*, and under drought, the mutant's flowering time was even earlier (Table 1). Notably, the mutant flowered earlier than *Col* under both WW and drought conditions (WW: 58.1 ± 1.06 vs. 58.6 ± 0.972 days; drought: 52.3 ± 3.58 vs. 68.1 ± 1.94 days), and unlike *Col*, the effect of drought on flowering time was not significant (*p* = 0.3981). Drought had significant effects on fitness‐related traits that reduced silique numbers, average silique length, and total silique length, while the GxTrt effects on fitness were generally not significant (Table 1, Tables S4–S6). *lsd1* plants had significantly lower inflorescence and rosette weights, thus lower aboveground biomass, compared to *Col* (Figure 2, Tables 1, S4–S6).

Both mutants and *Col* had increased WUE under drought compared to WW conditions (Figure 2), which was supported by significant treatment effects in LMMs that include *Col* and each mutant (p_*wrky38*_ = 9.33e‐18, p_*lsd1*_ = 1.15e‐6). The genotype effect of *WRKY38* knockout also significantly increased its WUE compared to *Col* (*p* = 8.81e‐8), while that of *lsd1* was not significant (*p* = 0.756; Figure 2). *wrky38* had an increased δ^15^N ratio under drought compared to the well‐watered condition, but its responses under both conditions were not substantially different from *Col* (*p*_*G*_ = 0.5010, *p*_Trt_ = 0.5575; Figure 2). *lsd1* mutants, however, had δ^15^N being lower under drought while slightly higher under WW compared to *Col* (*p*_*G*_ = 0.0387, *p*_GxTrt_ = 4.22e‐4), suggesting a possible nitrogen source shift under drought. C: N ratios had been overall reduced under drought, probably as a result of reduced biomass under drought (Figure 2).

### 3.3 Comparing Candidates From Different GEA Approaches

We compared each approach in identifying genes that influence fitness and performance traits under drought, potentially indicating their role in drought adaptation. Among the 40 top‐candidate genes from the original studies, 12, 12, 8, 6, and 7 genes were identified through RDA, partial RDA, univariate associations, the combined common garden approach with aridity index, and the combined common garden approach with monthly growing season precipitation, respectively (Table S1). Four genes were identified as top candidates by multiple approaches (Table S1). None of the knockout lines of these four genes had a significant genotype effect at FDR = 0.05 (Table S5).

RDA and partial RDA were the methods that identified the greatest number of significant genes, with each approach having 4 and 3 out of 12 genes exhibiting significant genotype effects at FDR = 0.05 across fitness and performance traits in the main drought screen. The combined GEA‐common garden approach followed, with 1 out of 6 and 1 out of 7 genes identified via association with aridity index and monthly precipitation showing significant genotype effects at FDR = 0.05. In contrast, none of the 8 genes identified by univariate associations showed significant genotype effects at FDR = 0.05, although 5 genes exhibited significant genotype effects at *α* = 0.05 before applying false discovery rate corrections.

### 3.4 Stable but Less Pronounced Fitness, Phenology, and Ecophysiological Responses of *wrky38* and *lsd1* Under Follow‐Up Mild Drought

**FIGURE 3:**
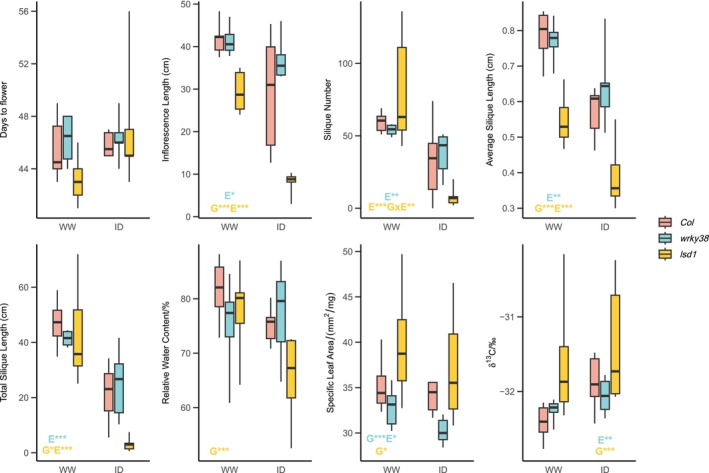
Traits measured in the follow‐up intermediate drought experiment of *Col*, *lsd1* (CS68738), and *wrky38* (CS864818). Text annotations on the box plots indicate the significance of genotype (G), treatment (E), and genotype‐treatment interaction (G × E) effects at *α* = 0.05, based on LMMs comparing *Col* and one mutant genotype. The text color denotes the mutant genotype. Significance levels are represented as follows: *, 0.01 ≤ *p* < 0.05; **, 0.001 ≤ *p* < 0.01; ***, *p* < 0.001.

Under the milder follow‐up drought (ID), *wrky38* and *lsd1* plants also exhibited similar but less pronounced responses compared to those observed in the main drought screen. The mild drought significantly reduced fitness and inflorescence length across both *Col* and the mutants, as determined by mixed linear models that included *Col* and one mutant (*wrky38* or *lsd1*). Specifically, the drought significantly reduced fitness and inflorescence length for both *wrky38* and *lsd1*, while flowering time was not significantly impacted (Figure 3, Table 2, Table S10). Plants of *wrky38* mutants did not exhibit significant genotype‐specific responses to drought compared to *Col* across the traits we measured (Table 2). However, the direction of *wrky38* differences was consistent with the earlier drought screen; e.g., *wrky38* had higher fitness and biomass than *Col* under drought, but the opposite under well‐watered conditions (Figure 3). In contrast, *lsd1* mutants showed significant genotype effects that reduced inflorescence length and total silique length and a significant GxE effect for silique number (Table 2). This indicates that *lsd1* mutants not only have reduced fitness under drought conditions, but the negative impact of drought was even more pronounced in *lsd1* mutants compared to *Col*.

**TABLE 2:** Summary statistics from mixed linear models (LMMs) assessing a subset of performance and fitness traits for *Col*, CS864818 (wrky38), and CS68738 (*lsd1*) under well‐watered and intermediate drought (ID) conditions during the follow‐up intermediate drought experiment. Each LMM independently analyses *Col* and one mutant (either *wrky38* or *lsd1*). *β* represents the estimated effect size, *p* represents the *p*‐value at *α* = 0.05, and subscripts indicate effects attributed to genotype (*G*), treatment (Trt), or genotype‐by‐treatment interaction (GxTrt). A negative *β*_*G*_ reflects a lower trait value in the mutant compared to *Col*, while a positive *β*_Trt_ reflects a lower trait value under ID compared to well‐watered conditions.

*Col*‐CS864818 (*wkry38*)	*β*_*G*_	*p*_G_	*β*_Trt_	*p*_Trt_	*β*_GxTrt_	*p*_GxTrt_
Days to flower (days)	0.50	0.1503	−0.33	0.9684	0.93	0.4626
Inflorescence length (cm)	7.75	0.2416	12.67	**0.0105**	−8.11	0.2551
Silique number	5.00	0.9632	26.67	**0.0023**	−10.50	0.4705
Total silique length (cm)	3.63	0.8752	25.68	**< 0.0001**	−9.23	0.2925
Specific leaf area (mm^2^/mg)	−3.80	**0.0005**	1.19	**0.0313**	1.43	0.4181

*Col*‐CS68738 (*lsd1*)	*β*_*G*_	*p*_G_	*β*_Trt_	*p*_Trt_	*β*_GxTrt_	*p*_GxTrt_
Days to flower (days)	1.42	0.7466	−0.33	0.1908	−3.63	0.1365
Silique number	−25.00	0.8293	26.67	**0.0007**	48.51	**0.0081**
Inflorescence length (cm)	−21.10	**< 0.0001**	12.67	**< 0.0001**	8.65	0.1897
Total silique length (cm)	−18.25	**0.0234**	25.68	**< 0.0001**	14.45	0.1378
Specific leaf area (mm^2^/mg)	3.11	0.0368	1.19	0.4479	1.41	0.6989

*Note:* *p*‐values less than 0.05 are presented in bold to indicate statistical significance.

During the 10 days of ecophysiological tracking, we built LMMs to compare the daily *g*_*sw*_ and Fv/fm of the two mutants with *Col*. The stomatal conductance (*g*_*sw*_) of all three genotypes exhibited fluctuations that were associated with the regular watering schedule (Figure S1). *wrky38* had potentially adaptive reduced *g*_*sw*_ compared to *Col* and under drought, with genotype effects significant during the initial days of tracking, when drought‐treated plants first experienced water limitation (9 days after treatment initiation; Table S11). A significant GxE interaction with drought was also observed (Table S11). The *lsd1* mutant also had reduced *g*_*sw*_ under drought, but the genotype effect was insignificant (Figure S1; Table S11). None of the Fv/fm values we measured suggested major oxidative stress (Figure S2), coinciding with Zivcak et al. (2013), indicating a mild drought. No genotype or treatment pattern was detected for Fv/fm.

The drought effect significantly reduced SLA when considering *Col* and *wrky38* but was not significant in the model including *Col* and *lsd1* (Figure 3, Table 2). Genotype effects on SLA were significant for both mutants, but *wrky38* had a lower SLA (potentially adaptive under drought) while *lsd1* had a higher SLA compared to *Col* (Figure 3, Table 2). RWC exhibited little variation between genotypes and treatments except for *lsd1* plants, which had a significant genotype effect resulting in lower RWC (Table S10). The δ^13^C response to treatment was generally lower in the follow‐up than in the main drought screen, indicating a milder drought in the follow‐up. The treatment effect on WUE was only significant for *wrky38* (*p* = 0.0029), whereas the genotype effect on WUE was only significant for *lsd1* (*p* = 3.7e‐4). No significant GxE effects were detected for SLA, RWC, or δ^13^C (Table S10).

### 3.5 Reduced Vegetative Growth or Fitness of *wrky38* and *lsd1* Under Overnight Freezing

**FIGURE 4:**
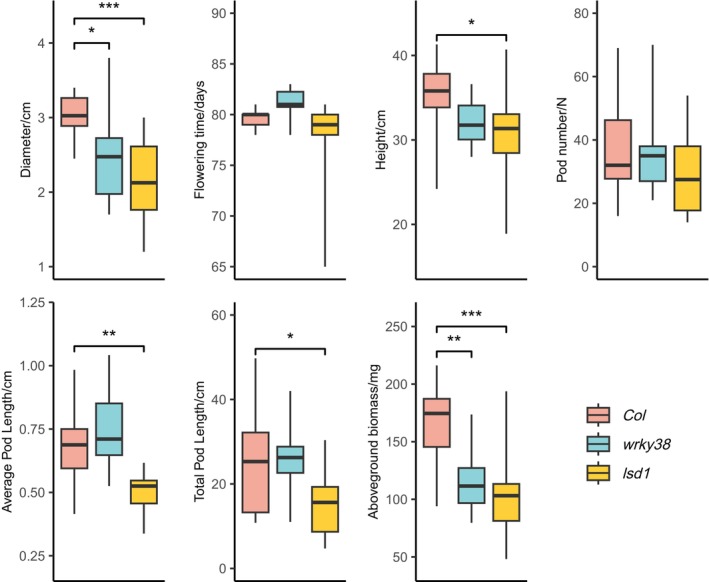
Traits measured in the follow‐up freezing experiment of *Col*, *lsd1* (CS68738), and *wrky38* (CS864818). Asterisks indicate significant trait differences between *Col* and each mutant based on Dunnett's test: *, 0.01 ≤ *p* < 0.05; **, 0.001 ≤ *p* < 0.01; ***, *p* < 0.001.

Treated with overnight freezing for 30 days, both *lsd1* and *wrky38* had significantly smaller diameters than *Col* (*p*_*wrky38*_ = 0.0184, *p*_*lsd1*_ = 3.7e‐4), representing their reduced vegetative growth under low temperatures (Figure 4). After returning to warmer temperatures, we found no variation in flowering time and silique number between *Col* and the two mutants. However, *lsd1* had a shorter inflorescence length (*p* = 0.0360), average silique length (*p* = 0.0023), and total silique length (*p* = 0.0218) than *Col*. Both *lsd1* and *wrky38* had smaller biomass than *Col* (*p*_*wrky38*_ = 0.0026, *p*_*lsd1*_ = 2.6e‐4), which might also be due to reduced vegetative growth. Overall, *lsd1* plants had reduced growth and fitness compared to *Col* under freezing, consistent with findings of Huang et al. (2010) that *lsd1* is chilling‐sensitive. Although natural putative LoF variants in *WRKY38* were strongly associated with warm winter temperatures (Lasky et al. 2018), *WRKY38* knockouts did not exhibit fitness differences compared to *Col*, though their vegetative growth was inhibited by overnight freezing compared to *Col*.

### 3.6 *WRKY38* Allelic Variation Associated With Climatic Moisture and Temperature

**FIGURE 5:**
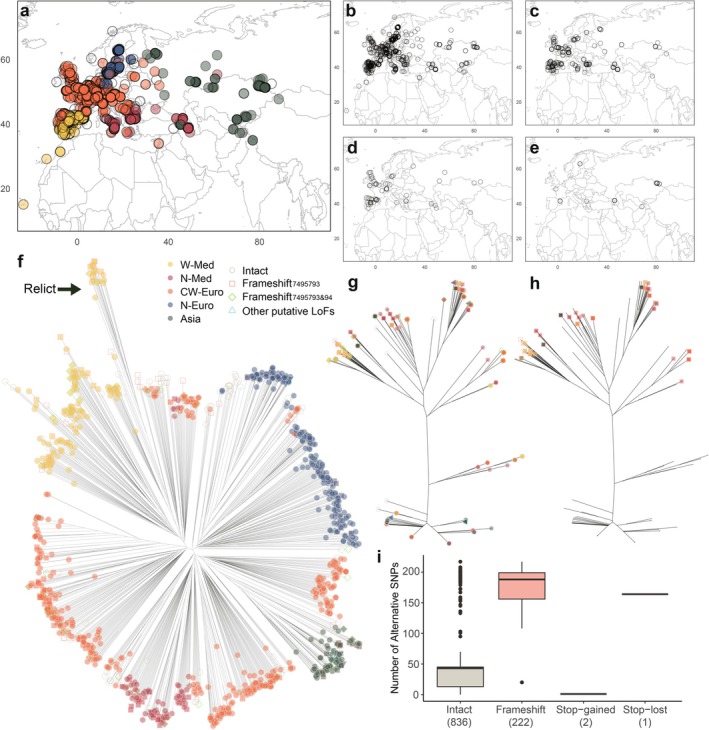
Natural variation of *WRKY38* putative LoF in the 1001 Genomes accessions. (a) distribution of native Arabidopsis accessions from 1001 Genomes. Colours indicate ADMIXTURE genetic clusters, originally defined by Alonso‐Blanco et al. (2016) and modified in this study. Accessions with clearly assigned genetic clusters are represented by filled icons in their respective cluster colours, while admixed accessions are shown with unfilled icons. (b–e) distributions of natural *WRKY38* putative functional variation: (b) distribution of accessions with intact *WRKY38*; (c) distribution of accessions with Frameshift_7495793_, the most common putative LoF variants of *WRKY38*; (d) distribution of accessions with Frameshift_7495793&94_, a mutation that restores the normal open reading frame; (e) distribution of accessions with other rare putative LoF variants of *WRKY38*. (f) *WRKY38* putative LoF variants on the genome‐wide NJ tree; the relict lineage (Alonso‐Blanco et al. 2016) was marked by an arrow. (g) *WRKY38* putative LoF variants on the NJ gene tree, symbols as in (f). (h) Frameshift_7495793_ on the NJ gene tree. (i) numbers of alternative SNPs in accessions with intact *WRKY38* and putative LoF variants within 10 kb around *WRKY38*. The number of accessions with each type of functional variation is indicated in parentheses.

To evaluate potential selection on *WRKY38* and its surrounding genomic region, we analysed SNP diversity patterns and examined allele associations indicating possible selection effects. The Tajima's D of the 5‐kb window containing the *WRKY38* gene was above 0 (Tajima's D = 1.429) and is above the average of Chromosome 5 (average Tajima's D = 0.660), but it did not significantly deviate from the distribution of Tajima's D across the chromosome (*z‐*score = 0.659; Figure S3). The overall *r* ^*2*^ between SNPs in 10 kb around *WRKY38* and the most common functional variant (Frameshift_7495793_) also indicated some haplotype structure, with the frameshift having elevated correlations (*r* ^*2*^ > 0.4) for SNPs located within 2 kb upstream of *WRKY38* and in the first 500 bp of the gene (also where the frameshift was located, maximum *r* ^*2*^ = 0.5104; Figure S4). However, this was not a dramatically long region of linkage that could suggest a recent sweep (cf. the strong correlation observed with chr4:10999188 and other SNPs in a 300 kb window around *LSD1*, Lee et al. 2017). Nevertheless, the putative LoF mutations carried large numbers of alternate SNP alleles across the locus, indicating some divergence and linkage (Figures 5i, S5). The haplotype structure at *WRKY38* can also be seen in the *WRKY38* gene neighbour‐joining tree. While the genome‐wide NJ tree shows a star‐like structure consistent with the recent expansion of Arabidopsis (Lee et al. 2017) with accessions clustered based on geography (Figure 5f), the *WRKY38* tree shows strong divergence between sequences with Frameshift_7495793_ versus putative intact *WRKY38* sequences (Figure 5g,h). This allelic variation is segregated across diverse lineages, including the relicts (Figure 5a–c,f). Coincidentally, the rare putative LoF variants were all unique to their lineages, suggesting recent origins (Figure 5f; Table S12). In contrast to the widespread Frameshift_7495793_, they might reflect newer local putative LoF mutations.

The gene tree of WRKY38 shows that alleles with the same putative LoF variants exhibit similar haplotype patterns, regardless of their genetic cluster (Figure 5g,h). For example, although most N‐Euro accessions are in the non‐Frameshift_7495793_ clade, all 12 N‐Euro accessions having Frameshift_7495793_ are in the Frameshift_7495793_ clade, overlapping with W‐Med, CW‐Euro, and Asian accessions carrying this allele. The only accession having Frameshift_7495794_ and the only one with Stop‐lost_7495609_ are both within the Frameshift_7495793_ clade, while the 6 accessions having Frameshift_7496041_ and 2 accessions having Stop‐gained_7496443_, which are grouped at the same tip on the gene tree, only occurred in the non‐Frameshift_7495793_ clade.

Inconsistency between the genome‐wide and *WRKY38* gene tree confirmed *WRKY38* allelic variation independent of population genetic structure. Next, we investigated whether this variation is associated with climatic factors, particularly moisture and drought‐related variables. We did not find significant correlations at *⍺* = 0.05 between the frequency of Frameshift_7495793_ (the most common putative LoF variant) or Frameshift_7495793&94_ (the variant that putatively restores the normal ORF) and the bioclimatic variables when considering kinship (Table S13), which coincided with the findings of Alonso‐Blanco et al. (2016) that almost no SNPs are significantly correlated with climate in environmental GWAS at FDR = 0.05 after considering population structure for 1001 Genomes accessions. Despite this, without accounting for kinship, significant differentiation was detected at *⍺* = 0.05 for 11, 12, and 4 bioclimatic variables between accessions with and without intact *WRKY38*, Frameshift_7495793_, and Frameshift_7495793&94_, respectively (Table S14). Among the significant associations, precipitation of the warmest quarter (bio18) had the lowest *p*‐values for both intact *WRKY38* (*p* = 6.8e‐10) and Frameshift_7495793_ (*p* = 8.8e‐9). Specifically, intact *WRKY38* occurred more frequently in regions with wet summers, while Frameshift_7495793_ was more common in hot and dry regions, suggesting the drought adaptation of *WRKY38* putative LoF. Frameshift_7495793&94_ (which has a net in‐frame effect) was most significantly correlated with higher isothermality (bio3; *p* = 3.7e‐4), followed by higher mean temperature of the wettest quarter (bio8; *p* = 6.4e‐4).

### 3.7 *LSD1* Allelic Variation and Expression Associated With Climatic Moisture and Temperature

**FIGURE 6:**
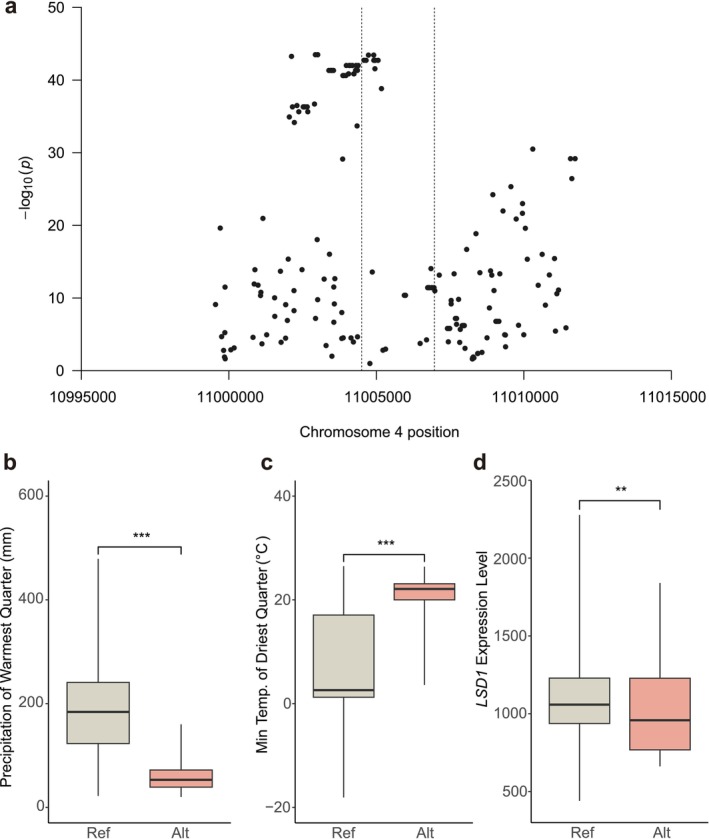
Genotype‐environment associations and *LSD1* expression. (a) Scanning the *LSD1* region to identify potential SNP‐environment associations using LMM within 10 kb. Each point represents the smallest *p*‐value from the 19 associations between each SNP and the 19 bioclimate variables. The *LSD1* gene region is shown between the dashed lines. These associations are meant to identify where in the locus variation is most strongly associated with climate. (b) The difference in precipitation of the warmest quarter (bio18) between reference and alternative alleles at a top climate‐associated SNP (Chr4, position 11,003,866). (c) The difference in the minimum temperature of the driest quarter (bio9) at an SNP (Chr4, position 11,003,866). (d) The difference in *LSD1* expression at an SNP (Chr4, position 11,003,866). (b–d) Asterisks indicate significant trait differences from *t*‐test: *, 0.01 ≤ *p* < 0.05; **, 0.001 ≤ *p* < 0.05; ***, *p* < 0.001.

Mixed models (GEMMA) detected strong correlations between SNPs within 10 kb around *LSD1*, particularly for SNPs within 1 kb upstream and the first 500 bp of *LSD1*, and multiple bioclimatic variables (Figure 6a). These SNPs, located in the promoter region of *LSD1*, may influence gene expression by altering transcription factor binding motifs (Shastry 2009; Wang et al. 2005). Thus, we hypothesised that these SNPs might contribute to local adaptation via changes in gene expression.

To validate the hypothesis, we picked a top climate‐associated SNP (Chr4, position 11,003,866) from the 1 kb upstream region of *LSD1* and tested for *LSD1* expression differences between ref/alt alleles from leaf tissue. This SNP had the strongest correlation, indicated by the lowest *p*‐value, with precipitation of the warmest quarter (bio18; wald‐*p* = 2.390e‐41; Figure 6b), followed by the minimum temperature of the driest quarter (bio9; wald‐*p* = 2.140e‐36; Figure 6c). We found that the alternative allele corresponded to significantly lower *LSD1* expression compared to the reference allele (*p* = 0.003; Figure 6d), suggesting a connection between lower *LSD1* expression and hot and dry climates.

Using linear models, we found that *LSD1* expression was significantly greater for genotypes from wetter summer climates (precipitation of the warmest quarter (*p* = 8.683e‐5) and precipitation of the wettest month (*p* = 0.039)). Across the 10 kb region around *LSD1*, 68 of the 201 SNPs were most strongly associated with bio18 (precipitation of the warmest quarter). Moreover, when limiting to SNPs within 1 kb upstream of *LSD1*, i.e., the region we thought to be linked with *LSD1*, the ratio became 25 of 35 SNPs. The results indicated that *LSD1* might play a role in local adaptation to moisture regimes by *cis*‐regulatory variation.

## 4 Discussion

Local adaptation is a major source of phenotypic variation within species, but traditional methods of identifying locally adapted genes are logistically challenging. Genotype‐environment associations (GEA) offer an easy‐to‐implement tool to generate hypotheses about genes involved in local adaptation, but most published results remain as untested hypotheses. Here, we screened 42 genes identified from genome‐wide GEA studies and found at least two that had effects on environmental responses and physiology linked to drought adaptation, suggesting potential local adaptations.

In our main drought experiment, among 42 potential drought‐adapted genes detected with 3 different GEA approaches, the tDNA knockout of *WRKY38* (detected with RDA, Lasky et al. 2012) exhibited a significant GxE effect on fitness‐related traits, and the *lsd1* mutant (gene detected with combined GEA‐common garden synthesis, Lasky et al. 2018) had a significant GxE effect on flowering time, which could potentially indicate variation in drought avoidance versus drought escape (Lovell et al. 2013). Coincidentally, these genes were both the top (#1) candidate genes with the strongest GEA in the studies used to select them. The physiological effect of *wrky38* identified in the follow‐up screening suggests mechanisms of drought avoidance via reduced stomatal conductance and SLA. Furthermore, the high frequency of putative natural loss‐of‐function alleles of *WRKY38* in Arabidopsis populations found in the drier, Mediterranean part of its range suggests potential local adaptation.

### 4.1 Genotype and Genotype‐By‐Environment Effects on Phenotypes

Although the genes screened had the largest effects in the original studies, the significant GxE effects for fitness in the main drought screen were no longer significant under the milder drought conditions of the follow‐up ID experiment. Several factors could impact the results. First, the milder conditions of the follow‐up drought experiment compared to the main drought screen may have reduced the G × E effect on fitness. Second, over‐simplified laboratory environments could lead to systematic bias in plant fitness (Anderson et al. 2013). In natural environments, drought conditions are usually coupled with elevated temperatures, a variable not accounted for in controlled experiments designed to isolate the effects of a single environmental factor. In the laboratory, when only a single environmental variable is changed between treatments, a conditional neutrality, that a gene has fitness advantages under one treatment and is neutral under the other, can be more common (Anderson et al. 2013). Lastly, the knockout alleles of a gene may not be the mechanism of local adaptation in nature. Local adaptation may also occur through *cis*‐regulatory effects on gene expression (Hämälä et al. 2020; Lasky et al. 2014) or changes in amino acid sequences. Therefore, when screening candidate genes for local adaptation, it is important to consider lines with significant genotype effects alongside those showing significant G × E effects for fitness. As G × E effects may not always be significant due to the factors discussed above, genotype effects may still capture environment‐associated differentiation.

Besides G × E effects on fitness, G × E effects on flowering time are also noteworthy when assessing local adaptation to drought stress, as early flowering is a critical drought‐escape strategy (Franks 2011; Kenney et al. 2014). Chong and Stinchcombe (2019) reported systematic directional effects of flowering time among randomly selected SALK lines. In their study, 11 out of 27 lines under long‐day conditions and 3 out of 27 lines under short‐day conditions exhibited significantly different flowering times compared to *Col*, with all showing delayed flowering. However, in our main drought screen, 21 out of 22 lines with significant genotype effects at ⍺ = 0.05, and 6 out of 7 lines with significant genotype effects at FDR = 0.05, exhibited accelerated flowering (Figure 1, Table S6). Among these, *wrky38* was the only line that showed delayed flowering (a potential drought avoidance strategy, Ludlow 1989). Since the genes we selected were candidates for drought adaptation, the accelerated flowering observed in these lines may indicate their potential for drought adaptation via escape (Ludlow 1989).

Though we only observed GxE for fitness in a small number of genes, evidence of associations between their natural functional variation and moisture‐related bioclimate variables suggested the underlying biological basis of potential local adaptation of loci identified by GEA. Setting up this screen was relatively easy due to the availability of existing tDNA resources, approaching an efficient way to identify and screen potential locally adapted genes in model organisms where such resources are available or relatively easily generated.

### 4.2 Comparison of Common GEA Approaches

Since the advent of GEA, a wide variety of methods have been proposed (reviewed by Rellstab et al. 2015). We tested candidates from three approaches here: multivariate ordination (RDA) of genotype and environment (Forester et al. 2018; Lasky et al. 2012), univariate climate mixed model associations (Lasky et al. 2014), and univariate climate models integrated with fitness data from multiple common gardens (Lasky et al. 2018). Even considering lines exhibiting significant genotype effects in addition to GxTrt effects, the number of positives remains too small to definitely compare these statistical GEA approaches. While Lotterhos (2023) recently criticized RDA as inappropriate for GEA due to a high false positive rate, the author used arbitrary and uncalibrated significance thresholds. By contrast, Figure S8 in Lotterhos (2023) showed that RDA was among the best methods based on the area under the precision–recall curve (AUC‐PR). Our results further support this, showing that RDA and partial RDA had the highest rate of genes exhibiting significant genotype effects at FDR = 0.05, followed by the combined common garden approach. In comparison, none of the tested genes identified through univariate associations showed significant genotype effects at FDR = 0.05.

We propose that using ranked GEA results is more informative than focusing on arbitrary significance thresholds; as mentioned previously, *WRKY38* and *LSD1* were the top‐ranked loci in their respective studies (RDA and combined GEA‐common garden mixed models, respectively; Lasky et al. 2012, 2018).

### 4.3 Potential Mechanisms of Drought Adaptation at *WRKY38* and *LSD1*

The two mutants presented opposite responses under drought. *wrky38* had a significantly smaller SLA when compared to *Col*, and when under drought, indicating how *wrky38* maintained stable RWC under well‐watered and drought conditions. *wrky38* mutants had reduced g_sw_, SLA, and leaf area (result not shown) under drought in the follow‐up experiment and increased WUE in response to drought during both experiments (Figures 2, 3), suggesting a drought‐avoidance strategy of natural *WRKY38* LoF genotypes. In contrast, *lsd1* had a larger SLA than *Col*, thus potentially more water loss (Niinemets 2001), which may result in its reduced RWC. Instead, *lsd1* mutants showed stable accelerated flowering under drought in our two drought experiments, indicating a drought‐escape strategy.

*WRKY38* negatively regulates plant basal pathogen defence by suppressing *PATHOGENESIS‐RELATED GENE 1* (*PR1*) expression, a defence gene induced by salicylic acid (SA) (Kim et al. 2008). Additionally, *WRKY38* is also involved in drought‐ and cold‐related responses in barley (Marè et al. 2004). Among Arabidopsis *WRKY* members that were also acting in the SA pathway for plant basal defence responses (Salinas et al. 2024), *AtWRKY54* and *AtWRKY70* were found to negatively impact osmotic tolerance by reducing stomatal closure (Li et al. 2013), while *AtWRKY46* was found to regulate light‐dependent stomatal opening in guard cells (Ding et al. 2014). Based on previous studies and our findings, we hypothesise that *WRKY38* inhibits stomatal closure. If so, the lower *g*_*sw*_ we found in knockouts could reduce water loss through the stomata and enhance fitness under drought.

In 1001 Genome accessions, natural *WRKY38* LoF alleles were strongly associated with lower July moisture compared to intact alleles. Despite this strong association, these putative LoF alleles were not fixed or entirely absent in any region. Our population genetics analyses revealed that LoF *WRKY38* exists across the entire distribution region of *A. thaliana* and in every lineage group (Figure 5a–f). We suspected that the LoF of *WRKY38* might have originated before the migration across Eurasia of *A. thaliana* and colonised drier regions during this process. In both drought experiments, *wrky38* generally had lower fitness compared to *Col* under well‐watered conditions. However, *wrky38* maintained relatively stable fitness under drought stress, while *Col* showed a substantial fitness decrease. The drought insensitivity of *wrky38* may explain how natural LoF *WRKY38* dominates drier regions during migration.

The absence of LSD1 protein in *lsd1* mutants promotes the accumulation of SA (Bernacki et al. 2021), which adversely affects plant development and reproduction while accelerating flowering in Arabidopsis (Salinas et al. 2024). These findings explained the consistent accelerated flowering behaviour observed across our two drought experiments. However, *lsd1* plants differed in their fitness responses to the long‐term water deficiency of our main drought screen and periodical moisture fluctuation in our follow‐up experiments (Figures 2, 3). Specifically, *lsd1* mutants had similar fitness to *Col* under well‐watered and drought conditions in the main experiment, but they were less fit than *Col* (i.e., the genotype effect was significant) in the follow‐up. Previous laboratory studies have shown that *lsd1* exhibited similar survival and seed production to the wild type under non‐lethal water deficiency. However, under lethal drought stress that caused complete mortality in wild‐type plants, *lsd1* demonstrated a significantly higher survival rate, albeit with reduced seed production. These findings suggest a trade‐off between survival and fecundity under severe stress in controlled laboratory conditions (Szechyńska‐Hebda et al. 2016; Wituszynska et al. 2013). When grown in the field, *lsd1* mutants did not exhibit seed yield differences compared to the wild type (Bernacki et al. 2019; Szechyńska‐Hebda et al. 2016; Wituszynska et al. 2013). This could explain our finding that the *LSD1* gene had lower expression in the hotter and drier regions, as reduced expression could similarly mitigate fitness trade‐offs, enhancing survival in stressful climates.

### 4.4 Freezing Responses of *wrky38* and *lsd1*

We conducted the freezing experiment because both genes may also be involved in adaptation to freezing stress. A SNP within the *WRKY38* coding region (Chr. 5, pos: 7496047) was the top candidate locus associated with the temperature of the coldest month in Lasky et al. (2018), and *LSD1* is associated with chilling sensitivity (Huang et al. 2010). Moreover, these two genes are both in the salicylic acid (SA) pathway that can be activated by cold stress (Miura and Tada 2014; Wu et al. 2019). *WRKY38* was positively regulated by SA (Kim et al. 2008), while *LSD1* conditionally regulated SA concentration in the presence of *ENHANCED DISEASE SUSCEPTIBILITY1* (*EDS1*) and *PHYTOALEXIN DEFICIENT4* (*PAD4*) (Bernacki et al. 2019; Szechyńska‐Hebda et al. 2016; Wituszynska et al. 2013).

*WRKY38* knockouts exhibited similar fitness to *Col* despite reduced vegetative growth after long‐term overnight freezing (Figure 4). Considering that *WRKY38* was originally identified through RDA (Lasky et al. 2012), this finding suggests a potential role in multivariate adaptation. The LoF allele might be advantageous in drier and warmer climates, while the functional allele might be more beneficial in cooler and wetter climates. *LSD1* knockouts had significantly reduced vegetative growth and fitness under freezing conditions, coincident with the findings of Huang et al. (2010). Both mutants exhibited some disadvantages under freezing conditions compared to *Col*, indicating possible links between the function of these genes and adaptation to cold climates. Although both drought and freezing conditions can limit water availability, and there is overlap in some molecular pathways or responses to each set of conditions (Kim et al. 2024), here our results suggest the drought‐adaptive variants are not cold‐adapted.

## 5 Conclusion

In this study, we tested the potential role of 44 t‐DNA knockout mutants of GEA‐identified genes in adaptation to drought stress using common garden experiments. While most mutants did not exhibit significant G × E effects for flowering time, performance, or fitness, two knockouts, *wrky38* and *lsd1*, demonstrated evidence of drought adaptation in both the main drought screen and follow‐up intermediate drought common gardens. Natural variation in the function or expression of these genes across a moisture gradient further supports the utility of GEA approaches for generating hypotheses, though further experiments are required to test hypotheses. Our findings highlight the promise of GEA methods for uncovering novel local adaptations to environmental stressors.

## Author Contributions

Y.L., C.M.L., and J.R.L. designed the study, C.M.L. and Y.L. performed experiments, Y.L. conducted data analyses, Y.L., E.H.L.‐P., and J.R.L. interpreted results, Y.L., C.M.L., and J.R.L. wrote the manuscript, and all authors edited and approved the manuscript.

## Disclosure

Benefit‐sharing statement: This study is based on publicly available genomic data (1001 Genomes Project) and trait data collected by the authors. All trait data and analysis scripts are shared in an open‐access repository. No additional benefit‐sharing obligations apply.

## Conflicts of Interest

The authors declare no conflicts of interest.

## Supporting information


**Figure S1:** mec70129‐sup‐0001‐FiguresS1‐S5.pdf.
**Figure S2:** mec70129‐sup‐0001‐FiguresS1‐S5.pdf.
**Figure S3:** mec70129‐sup‐0001‐FiguresS1‐S5.pdf.
**Figure S4:** mec70129‐sup‐0001‐FiguresS1‐S5.pdf.
**Figure S5:** mec70129‐sup‐0001‐FiguresS1‐S5.pdf.

**Table S1:** mec70129‐sup‐0002‐TablesS1‐S9.xlsx.
**Table S2:** mec70129‐sup‐0002‐TablesS1‐S9.xlsx.
**Table S3:** mec70129‐sup‐0002‐TablesS1‐S9.xlsx.
**Table S4:** mec70129‐sup‐0002‐TablesS1‐S9.xlsx.
**Table S5:** mec70129‐sup‐0002‐TablesS1‐S9.xlsx.
**Table S6:** mec70129‐sup‐0002‐TablesS1‐S9.xlsx.
**Table S7:** mec70129‐sup‐0002‐TablesS1‐S9.xlsx.
**Table S8:** mec70129‐sup‐0002‐TablesS1‐S9.xlsx.
**Table S9:** mec70129‐sup‐0002‐TablesS1‐S9.xlsx.

## Data Availability

Trait data in this study and the analysis scripts are available from Dryad at https://doi.org/10.5061/dryad.qrfj6q5s4. Genomic data were obtained from the 1001 Genomes Project (https://1001genomes.org) and have been published previously (Alonso‐Blanco et al. 2016).

## References

[mec70129-bib-0001] Alonso, J. M. , A. N. Stepanova , T. J. Leisse , et al. 2003. “Genome‐Wide Insertional Mutagenesis of *Arabidopsis thaliana* .” Science 301: 653–657. 10.1126/science.1086391. 12893945

[mec70129-bib-0002] Alonso‐Blanco, C. , J. Andrade , C. Becker , et al. 2016. “1,135 Genomes Reveal the Global Pattern of Polymorphism in *Arabidopsis thaliana* .” Cell 166: 481–491. 10.1016/j.cell.2016.05.063. 27293186 PMC4949382

[mec70129-bib-0003] Anderson, J. T. , C.‐R. Lee , C. A. Rushworth , R. I. Colautti , and T. Mitchell‐Olds . 2013. “Genetic Trade‐Offs and Conditional Neutrality Contribute to Local Adaptation.” Molecular Ecology 22: 699–708. 10.1111/j.1365-294X.2012.05522.x. 22420446 PMC3492549

[mec70129-bib-0004] Benjamini, Y. , and D. Yekutieli . 2001. “The Control of the False Discovery Rate in Multiple Testing Under Dependency.” Annals of Statistics 29: 1165–1188.

[mec70129-bib-0005] Bernacki, M. J. , W. Czarnocka , A. Rusaczonek , et al. 2019. “LSD1‐, EDS1‐ and PAD4‐Dependent Conditional Correlation Among Salicylic Acid, Hydrogen Peroxide, Water Use Efficiency and Seed Yield in *Arabidopsis thaliana* .” Physiologia Plantarum 165: 369–382. 10.1111/ppl.12863. 30461017

[mec70129-bib-0006] Bernacki, M. J. , A. Rusaczonek , W. Czarnocka , and S. Karpiński . 2021. “Salicylic Acid Accumulation Controlled by LSD1 Is Essential in Triggering Cell Death in Response to Abiotic Stress.” Cells 10: 962. 10.3390/cells10040962. 33924244 PMC8074770

[mec70129-bib-0007] Betancourt, N. J. , S. Rajpurohit , E. Durmaz , et al. 2021. “Allelic Polymorphism at Foxo Contributes to Local Adaptation in *Drosophila melanogaster* .” Molecular Ecology 30: 2817–2830. 10.1111/mec.15939. 33914989 PMC8693798

[mec70129-bib-0008] Caicedo, A. L. , C. Richards , I. M. Ehrenreich , and M. D. Purugganan . 2009. “Complex Rearrangements Lead to Novel Chimeric Gene Fusion Polymorphisms at the *Arabidopsis thaliana MAF2‐5* Flowering Time Gene Cluster.” Molecular Biology and Evolution 26: 699–711. 10.1093/molbev/msn300. 19139056

[mec70129-bib-0009] Capblancq, T. , S. Lachmuth , M. C. Fitzpatrick , and S. R. Keller . 2023. “From Common Gardens to Candidate Genes: Exploring Local Adaptation to Climate in Red Spruce.” New Phytologist 237: 1590–1605. 10.1111/nph.18465. 36068997 PMC10092705

[mec70129-bib-0010] Chong, V. K. , and J. R. Stinchcombe . 2019. “Evaluating Population Genomic Candidate Genes Underlying Flowering Time in *Arabidopsis thaliana* Using T‐DNA Insertion Lines.” Journal of Heredity 110: 445–454. 10.1093/jhered/esz026. 31158286

[mec70129-bib-0011] Cingolani, P. , A. Platts , L. L. Wang , et al. 2012. “A Program for Annotating and Predicting the Effects of Single Nucleotide Polymorphisms, SnpEff: SNPs in the Genome of *Drosophila melanogaster* Strain w1118; Iso‐2; Iso‐3.” Fly (Austin) 6: 80–92. 10.4161/fly.19695. 22728672 PMC3679285

[mec70129-bib-0012] Clausen, J. , D. D. Keck , and W. M. Hiesey . 1941. “Regional Differentiation in Plant Species.” American Naturalist 75: 231–250.

[mec70129-bib-0013] Coop, G. , D. Witonsky , A. Di Rienzo , and J. K. Pritchard . 2010. “Using Environmental Correlations to Identify Loci Underlying Local Adaptation.” Genetics 185: 1411–1423. 10.1534/genetics.110.114819. 20516501 PMC2927766

[mec70129-bib-0014] Cortés, A. J. , F. López‐Hernández , and M. W. Blair . 2022. “Genome–Environment Associations, an Innovative Tool for Studying Heritable Evolutionary Adaptation in Orphan Crops and Wild Relatives.” Frontiers in Genetics 13: 910386. 10.3389/fgene.2022.910386. 35991553 PMC9389289

[mec70129-bib-0015] Crossa, J. , P. Pérez‐Rodríguez , J. Cuevas , et al. 2017. “Genomic Selection in Plant Breeding: Methods, Models, and Perspectives.” Trends in Plant Science 22: 961–975. 10.1016/j.tplants.2017.08.011. 28965742

[mec70129-bib-0016] Ding, Z. J. , J. Y. Yan , X. Y. Xu , et al. 2014. “Transcription Factor WRKY46 Regulates Osmotic Stress Responses and Stomatal Movement Independently in Arabidopsis.” Plant Journal 79: 13–27. 10.1111/tpj.12538. 24773321

[mec70129-bib-0017] Dittberner, H. , A. Korte , T. Mettler‐Altmann , A. P. M. Weber , G. Monroe , and J. de Meaux . 2018. “Natural Variation in Stomata Size Contributes to the Local Adaptation of Water‐Use Efficiency in *Arabidopsis thaliana* .” Molecular Ecology 27: 4052–4065. 10.1111/mec.14838. 30118161 PMC7611081

[mec70129-bib-0018] El‐Soda, M. , W. Kruijer , M. Malosetti , M. Koornneef , and M. G. M. Aarts . 2015. “Quantitative Trait Loci and Candidate Genes Underlying Genotype by Environment Interaction in the Response of *Arabidopsis thaliana* to Drought.” Plant, Cell & Environment 38: 585–599. 10.1111/pce.12418. 25074022

[mec70129-bib-0019] Endler, J. A. 1973. “Gene Flow and Population Differentiation.” Science 179: 243–250. 10.1126/science.179.4070.243. 4630250

[mec70129-bib-0020] Exposito‐Alonso, M. , F. Vasseur , W. Ding , G. Wang , H. A. Burbano , and D. Weigel . 2018. “Genomic Basis and Evolutionary Potential for Extreme Drought Adaptation in *Arabidopsis thaliana* .” Nature Ecology & Evolution 2: 352–358. 10.1038/s41559-017-0423-0. 29255303 PMC5777624

[mec70129-bib-0021] Fick, S. E. , and R. J. Hijmans . 2017. “WorldClim 2: New 1‐Km Spatial Resolution Climate Surfaces for Global Land Areas.” International Journal of Climatology 37: 4302–4315. 10.1002/joc.5086.

[mec70129-bib-0022] Forester, B. R. , J. R. Lasky , H. H. Wagner , and D. L. Urban . 2018. “Comparing Methods for Detecting Multilocus Adaptation With Multivariate Genotype–Environment Associations.” Molecular Ecology 27: 2215–2233. 10.1111/mec.14584. 29633402

[mec70129-bib-0023] Franks, S. J. 2011. “Plasticity and Evolution in Drought Avoidance and Escape in the Annual Plant *Brassica rapa* .” New Phytologist 190: 249–257. 10.1111/j.1469-8137.2010.03603.x. 21210818

[mec70129-bib-0024] Gehan, M. A. , S. Park , S. J. Gilmour , C. An , C.‐M. Lee , and M. F. Thomashow . 2015. “Natural Variation in the C‐Repeat Binding Factor Cold Response Pathway Correlates With Local Adaptation of Arabidopsis Ecotypes.” Plant Journal 84: 682–693. 10.1111/tpj.13027. 26369909

[mec70129-bib-0025] Hager, E. R. , O. S. Harringmeyer , T. B. Wooldridge , et al. 2022. “A Chromosomal Inversion Contributes to Divergence in Multiple Traits Between Deer Mouse Ecotypes.” Science 377: 399–405. 10.1126/science.abg0718. 35862520 PMC9571565

[mec70129-bib-0026] Hämälä, T. , A. J. Gorton , D. A. Moeller , and P. Tiffin . 2020. “Pleiotropy Facilitates Local Adaptation to Distant Optima in Common Ragweed ( *Ambrosia artemisiifolia* ).” PLoS Genetics 16: e1008707. 10.1371/journal.pgen.1008707. 32210431 PMC7135370

[mec70129-bib-0027] Hancock, A. M. , B. Brachi , N. Faure , et al. 2011. “Adaptation to Climate Across the *Arabidopsis thaliana* Genome.” Science 334: 83–86. 10.1126/science.1209244. 21980108

[mec70129-bib-0028] Huang, X. , Y. Li , X. Zhang , J. Zuo , and S. Yang . 2010. “The Arabidopsis LSD1 Gene Plays an Important Role in the Regulation of Low Temperature‐Dependent Cell Death.” New Phytologist 187: 301–312. 10.1111/j.1469-8137.2010.03275.x. 20456049

[mec70129-bib-0029] Kang, H. M. , N. A. Zaitlen , C. M. Wade , et al. 2008. “Efficient Control of Population Structure in Model Organism Association Mapping.” Genetics 178: 1709–1723. 10.1534/genetics.107.080101. 18385116 PMC2278096

[mec70129-bib-0030] Kawakatsu, T. , S. C. Huang , F. Jupe , et al. 2016. “Epigenomic Diversity in a Global Collection of *Arabidopsis thaliana* Accessions.” Cell 166: 492–505. 27419873 10.1016/j.cell.2016.06.044 PMC5172462

[mec70129-bib-0081] Kawecki, T. J. , and D. Ebert . 2004. “Conceptual Issues in Local Adaptation.” Ecology Letters 7, no. 12: 1225–1241. 10.1111/j.1461-0248.2004.00684.x.

[mec70129-bib-0031] Kenney, A. M. , J. K. McKay , J. H. Richards , and T. E. Juenger . 2014. “Direct and Indirect Selection on Flowering Time, Water‐Use Efficiency (WUE, δ13C), and WUE Plasticity to Drought in *Arabidopsis thaliana* .” Ecology and Evolution 4: 4505–4521. 10.1002/ece3.1270. 25512847 PMC4264900

[mec70129-bib-0032] Kim, J.‐S. , S. Kidokoro , K. Yamaguchi‐Shinozaki , and K. Shinozaki . 2024. “Regulatory Networks in Plant Responses to Drought and Cold Stress.” Plant Physiology 195: 170–189. 10.1093/plphys/kiae105. 38514098 PMC11060690

[mec70129-bib-0033] Kim, K. C. , Z. Lai , B. Fan , and Z. Chen . 2008. “Arabidopsis WRKY38 and WRKY62 Transcription Factors Interact With Histone Deacetylase 19 in Basal Defense.” Plant Cell 20: 2357–2371. 10.1105/tpc.107.055566. 18776063 PMC2570728

[mec70129-bib-0034] Lasky, J. R. , D. L. Des Marais , D. B. Lowry , et al. 2014. “Natural Variation in Abiotic Stress Responsive Gene Expression and Local Adaptation to Climate in *Arabidopsis thaliana* .” Molecular Biology and Evolution 31: 2283–2296. 10.1093/molbev/msu170. 24850899 PMC4137704

[mec70129-bib-0035] Lasky, J. R. , D. L. Des Marais , J. K. McKAY , J. H. Richards , T. E. Juenger , and T. H. Keitt . 2012. “Characterizing Genomic Variation of *Arabidopsis thaliana*: The Roles of Geography and Climate.” Molecular Ecology 21: 5512–5529. 10.1111/j.1365-294X.2012.05709.x. 22857709

[mec70129-bib-0036] Lasky, J. R. , B. R. Forester , and M. Reimherr . 2018. “Coherent Synthesis of Genomic Associations With Phenotypes and Home Environments.” Molecular Ecology Resources 18: 91–106. 10.1111/1755-0998.12714. 28861950

[mec70129-bib-0037] Lasky, J. R. , E. B. Josephs , and G. P. Morris . 2023. “Genotype–Environment Associations to Reveal the Molecular Basis of Environmental Adaptation.” Plant Cell 35: 125–138. 10.1093/plcell/koac267. 36005926 PMC9806588

[mec70129-bib-0038] Lawrence‐Paul, E. H. , J. Janakiraman , M. R. Lawrence‐Paul , R. Ben‐Zeev , A. Penn , and J. R. Lasky . 2025. “It's All in the Timing: Vegetative Phase Change Alters Selection Under Drought and Contributes to Local Adaptation.” bioRxiv. 2025.05.13.653704. 10.1101/2025.05.13.653704.

[mec70129-bib-0039] Lawrence‐Paul, E. H. , R. S. Poethig , and J. R. Lasky . 2023. “Vegetative Phase Change Causes Age‐Dependent Changes in Phenotypic Plasticity.” New Phytologist 240: 613–625. 10.1111/nph.19174. 37571856 PMC10551844

[mec70129-bib-0040] Lee, C.‐R. , H. Svardal , A. Farlow , et al. 2017. “On the Post‐Glacial Spread of Human Commensal *Arabidopsis thaliana* .” Nature Communications 8: 14458. 10.1038/ncomms14458. PMC5309843 28181519

[mec70129-bib-0041] Lee, G. , B. J. Sanderson , T. J. Ellis , et al. 2024. “A Large‐Effect Fitness Trade‐Off Across Environments Is Explained by a Single Mutation Affecting Cold Acclimation.” Proceedings of the National Academy of Sciences of the United States of America 121: e2317461121. 10.1073/pnas.2317461121. 38289961 PMC10861903

[mec70129-bib-0042] Lenth, R. 2025. “*Emmeans*: Estimated Marginal Means, Aka Least‐Squares Means. R Package Version 1.11.2–80001.” https://rvlenth.github.io/emmeans/.

[mec70129-bib-0043] Li, J. , S. Besseau , P. Törönen , et al. 2013. “Defense‐Related Transcription Factors WRKY70 and WRKY54 Modulate Osmotic Stress Tolerance by Regulating Stomatal Aperture in Arabidopsis.” New Phytologist 200: 457–472. 10.1111/nph.12378. 23815736 PMC4284015

[mec70129-bib-0044] Lorts, C. M. , and J. R. Lasky . 2020. “Competition × Drought Interactions Change Phenotypic Plasticity and the Direction of Selection on Arabidopsis Traits.” New Phytologist 227: 1060–1072. 10.1111/nph.16593. 32267968

[mec70129-bib-0045] Lotterhos, K. E. 2023. “The Paradox of Adaptive Trait Clines With Nonclinal Patterns in the Underlying Genes.” Proceedings of the National Academy of Sciences of the United States of America 120: e2220313120. 10.1073/pnas.2220313120. 36917658 PMC10041142

[mec70129-bib-0046] Lovell, J. T. , T. E. Juenger , S. D. Michaels , et al. 2013. “Pleiotropy of FRIGIDA Enhances the Potential for Multivariate Adaptation.” Proceedings of the Royal Society B: Biological Sciences 280: 20131043. 10.1098/rspb.2013.1043. PMC3774242 23698015

[mec70129-bib-0047] Ludlow, M. M. 1989. “Strategies of Response to Water Stress.” In Structural and Functional Responses to Environmental Stresses, edited by K. H. Kreeb , H. Richter , and T. M. Hinckley , 269–281. SPB Academic.

[mec70129-bib-0049] Marè, C. , E. Mazzucotelli , C. Crosatti , E. Francia , A. M. Stanca , and L. Cattivelli . 2004. “Hv‐WRKY38: A New Transcription Factor Involved in Cold‐ and Drought‐Response in Barley.” Plant Molecular Biology 55: 399–416. 10.1007/s11103-004-0906-7. 15604689

[mec70129-bib-0050] Miura, K. , and Y. Tada . 2014. “Regulation of Water, Salinity, and Cold Stress Responses by Salicylic Acid.” Frontiers in Plant Science 5: 4. 24478784 10.3389/fpls.2014.00004 PMC3899523

[mec70129-bib-0051] Monroe, J. G. , T. Powell , N. Price , et al. 2018. “Drought Adaptation in *Arabidopsis thaliana* by Extensive Genetic Loss‐Of‐Function.” eLife 7: e41038. 10.7554/eLife.41038. 30520727 PMC6326724

[mec70129-bib-0052] Niinemets, Ü. 2001. “Global‐Scale Climatic Controls of Leaf Dry Mass Per Area, Density, and Thickness in Trees and Shrubs.” Ecology 82: 453–469. 10.1890/0012-9658(2001)082[0453:GSCCOL]2.0.CO;2.

[mec70129-bib-0053] Nyine, M. , D. Davidson , E. Adhikari , et al. 2025. “Genomic Signals of Ecogeographic Adaptation in a Wild Relative Are Associated With Improved Wheat Performance Under Drought Stress.” Genome Biology 26: 35. 10.1186/s13059-025-03500-1. 39985084 PMC11844086

[mec70129-bib-0054] Paradis, E. , and K. Schliep . 2019. “Ape 5.0: An Environment for Modern Phylogenetics and Evolutionary Analyses in R.” Bioinformatics 35: 526–528. 30016406 10.1093/bioinformatics/bty633

[mec70129-bib-0055] Pisupati, R. , I. Reichardt , Ü. Seren , et al. 2017. “Verification of Arabidopsis Stock Collections Using SNPmatch, a Tool for Genotyping High‐Plexed Samples.” Scientific Data 4: 170184. 10.1038/sdata.2017.184. 29257129 PMC5744633

[mec70129-bib-0056] Purcell, S. , B. Neale , K. Todd‐Brown , et al. 2007. “PLINK: A Tool Set for Whole‐Genome Association and Population‐Based Linkage Analyses.” American Journal of Human Genetics 81: 559–575. 17701901 10.1086/519795 PMC1950838

[mec70129-bib-0057] Ralph, P. L. , and G. Coop . 2015. “Convergent Evolution During Local Adaptation to Patchy Landscapes.” PLoS Genetics 11: e1005630. 10.1371/journal.pgen.1005630. 26571125 PMC4646681

[mec70129-bib-0058] Ratcliffe, O. J. , R. W. Kumimoto , B. J. Wong , and J. L. Riechmann . 2003. “Analysis of the Arabidopsis MADS AFFECTING FLOWERING Gene Family: MAF2 Prevents Vernalization by Short Periods of Cold [W].” Plant Cell 15: 1159–1169. 10.1105/tpc.009506. 12724541 PMC153723

[mec70129-bib-0059] Rellstab, C. , F. Gugerli , A. J. Eckert , A. M. Hancock , and R. Holderegger . 2015. “A Practical Guide to Environmental Association Analysis in Landscape Genomics.” Molecular Ecology 24: 4348–4370. 10.1111/mec.13322. 26184487

[mec70129-bib-0060] Salinas, P. , S. Velozo , and A. Herrera‐Vásquez . 2024. “Salicylic Acid Accumulation: Emerging Molecular Players and Novel Perspectives on Plant Development and Nutrition.” Journal of Experimental Botany 76: erae309. 10.1093/jxb/erae309. PMC12066125 39028261

[mec70129-bib-0061] Savolainen, O. , M. Lascoux , and J. Merilä . 2013. “Ecological Genomics of Local Adaptation.” Nature Reviews. Genetics 14: 807–820. 10.1038/nrg3522. 24136507

[mec70129-bib-0062] Shastry, B. S. 2009. “SNPs: Impact on Gene Function and Phenotype.” In Single Nucleotide Polymorphisms: Methods and Protocols, edited by A. A. Komar , 3–22. Humana Press. 10.1007/978-1-60327-411-1_1. 19768584

[mec70129-bib-0063] Signorell, A. , K. Aho , A. Alfons , et al. 2023. “DescTools: Tools for Descriptive Statistics.” R Package Version 0.99. 26. Compr. R. Arch Netw. 289–291.

[mec70129-bib-0064] Sinnwell, J. P. , T. M. Therneau , and D. J. Schaid . 2014. “The Kinship2 R Package for Pedigree Data.” Human Heredity 78: 91–93. 25074474 10.1159/000363105 PMC4154601

[mec70129-bib-0065] Soudi, S. , M. Jahani , M. Todesco , et al. 2023. “Repeatability of Adaptation in Sunflowers Reveals That Genomic Regions Harbouring Inversions Also Drive Adaptation in Species Lacking an Inversion.” eLife 12: RP88604. 10.7554/eLife.88604. 38095362 PMC10721221

[mec70129-bib-0066] Szechyńska‐Hebda, M. , W. Czarnocka , M. Hebda , and S. Karpiński . 2016. “PAD4, LSD1 and EDS1 Regulate Drought Tolerance, Plant Biomass Production, and Cell Wall Properties.” Plant Cell Reports 35: 527–539. 10.1007/s00299-015-1901-y. 26754794

[mec70129-bib-0067] Tergemina, E. , S. Ansari , D. E. Salt , and A. M. Hancock . 2025. “Multiple Independent MGR5 Alleles Contribute to a Clinal Pattern in Leaf Magnesium Across the Distribution of *Arabidopsis thaliana* .” New Phytologist 246: 1861–1874. 10.1111/nph.70069. 40125608 PMC12018779

[mec70129-bib-0068] Therneau, T. M. , and M. T. M. Therneau . 2015. Package ‘Coxme’. R Package Version 2.

[mec70129-bib-0069] Tigano, A. , and V. L. Friesen . 2016. “Genomics of Local Adaptation With Gene Flow.” Molecular Ecology 25: 2144–2164. 10.1111/mec.13606. 26946320

[mec70129-bib-0070] Wadgymar, S. M. , M. L. DeMarche , E. B. Josephs , S. N. Sheth , and J. T. Anderson . 2022. “Local Adaptation: Causal Agents of Selection and Adaptive Trait Divergence.” Annual Review of Ecology, Evolution, and Systematics 53: 87–111. 10.1146/annurev-ecolsys-012722-035231. PMC10544833 37790997

[mec70129-bib-0071] Wang, X. , D. J. Tomso , X. Liu , and D. A. Bell . 2005. “Single Nucleotide Polymorphism in Transcriptional Regulatory Regions and Expression of Environmentally Responsive Genes.” Toxicology and Applied Pharmacology 207: 84–90. 10.1016/j.taap.2004.09.024. 16002116

[mec70129-bib-0072] Whiting, J. R. , T. R. Booker , C. Rougeux , et al. 2024. “The Genetic Architecture of Repeated Local Adaptation to Climate in Distantly Related Plants.” Nature Ecology & Evolution 8: 1933–1947. 10.1038/s41559-024-02514-5. 39187610 PMC11461274

[mec70129-bib-0074] Wituszynska, W. , I. Slesak , S. Vanderauwera , et al. 2013. “Lesion Simulating Disease1, Enhanced Disease Susceptibility1, and Phytoalexin Deficient4 Conditionally Regulate Cellular Signaling Homeostasis, Photosynthesis, Water Use Efficiency, and Seed Yield in Arabidopsis.” Plant Physiology 161: 1795–1805. 10.1104/pp.112.208116. 23400705 PMC3613456

[mec70129-bib-0075] Wu, Z. , S. Han , H. Zhou , et al. 2019. “Cold Stress Activates Disease Resistance in *Arabidopsis thaliana* Through a Salicylic Acid Dependent Pathway.” Plant, Cell & Environment 42: 2645–2663. 10.1111/pce.13579. 31087367

[mec70129-bib-0076] Yeaman, S. , and M. C. Whitlock . 2011. “The Genetic Architecture of Adaptation Under Migration–Selection Balance.” Evolution 65: 1897–1911. 10.1111/j.1558-5646.2011.01269.x. 21729046

[mec70129-bib-0077] Yim, C. , E. S. Bellis , V. L. DeLeo , D. Gamba , R. Muscarella , and J. R. Lasky . 2024. “Climate Biogeography of *Arabidopsis thaliana* : Linking Distribution Models and Individual Variation.” Journal of Biogeography 51: 560–574. 10.1111/jbi.14737. 38596256 PMC11000247

[mec70129-bib-0078] Zhao, M. , M. Li , M. Huang , et al. 2023. “The Cysteine‐Rich Receptor‐Like Kinase CRK4 Contributes to the Different Drought Stress Response Between Columbia and Landsberg Erecta.” Plant, Cell & Environment 46: 3258–3272. 10.1111/pce.14665. 37427814

[mec70129-bib-0079] Zivcak, M. , M. Brestic , Z. Balatova , et al. 2013. “Photosynthetic Electron Transport and Specific Photoprotective Responses in Wheat Leaves Under Drought Stress.” Photosynthesis Research 117: 529–546. 10.1007/s11120-013-9885-3. 23860828

[mec70129-bib-0080] Zoubarev, A. , K. M. Hamer , K. D. Keshav , et al. 2012. “Gemma: A Resource for the Reuse, Sharing and Meta‐Analysis of Expression Profiling Data.” Bioinformatics 28: 2272–2273. 10.1093/bioinformatics/bts430. 22782548 PMC3426847

