# Supplementary material for: Experimental Validation of Genome‐Environment Associations in Arabidopsis
Source: Mol Ecol. 2025 Oct 6;34(21):e70129. doi: 10.1111/mec.70129 (PMC12573728; doi:10.1111/mec.70129)
Supplement: Supplementary file 1 — Figure S1: mec70129‐sup‐0001‐FiguresS1‐S5.pdf. Figure S2: mec70129‐sup‐0001‐FiguresS1‐S5.pdf. Figure S3: mec70129‐sup‐0001‐FiguresS1‐S5.pdf. Figure S4: mec70129‐sup‐0001‐FiguresS1‐S5.pdf. Figure S5: mec70129‐sup‐0001‐FiguresS1‐S5.pdf. [file MEC-34-e70129-s001.pdf]

Supplemental Information for:

Experimental validation of genome-environment associations in Arabidopsis

Yuxin Luo, Claire M. Lorts, Erica H. Lawrence-Paul, Jesse R. Lasky

Table of Contents in Supplemental Information.zip:

**Figure S1-S5 and captions**	Page 2-7
**Table S1-S14 and captions**	SupplementaryTables.xlsx

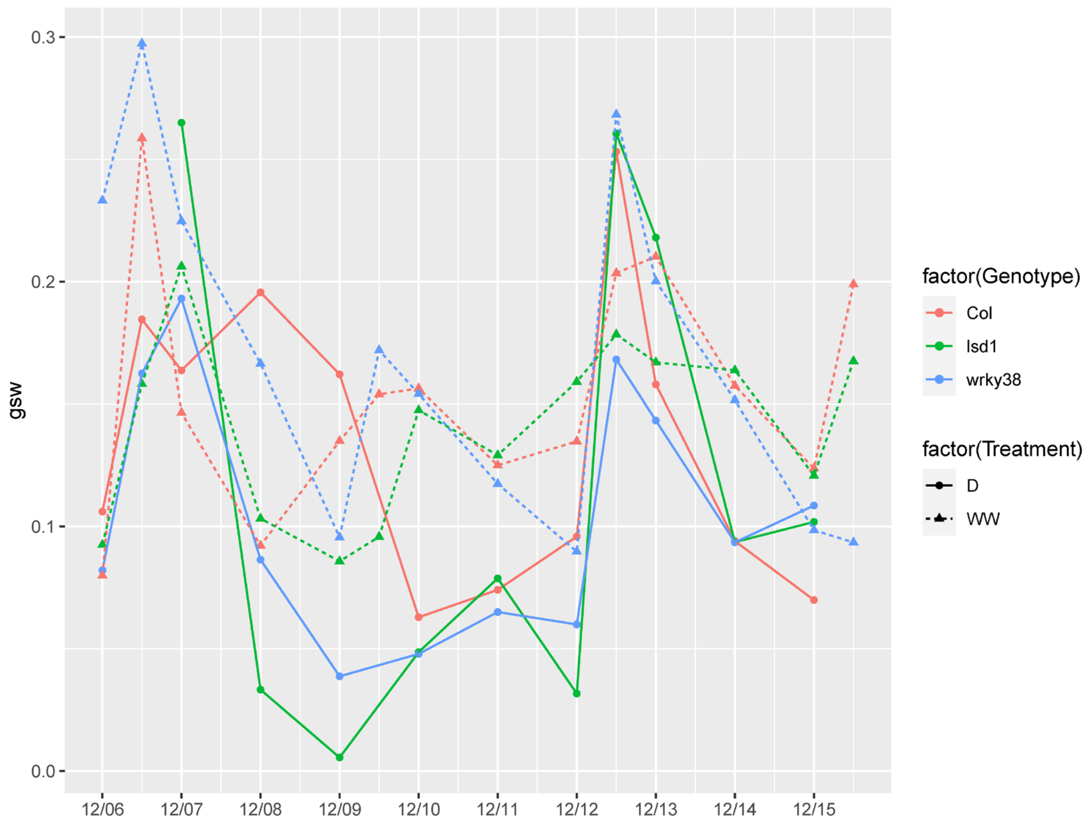

Figure S1 Stomatal conductance (g_{sw}) changes across 10 consecutive days in *Col*, *wrky38*, and *lsd1* measured with LI-COR 600. Measurements were taken at ~1.5 h before lights were off. On the watering day, two g_{sw} measurements were taken right before and ~0.5h after watering. Each point represents mean g_{sw} from 3-4 plants of the same genotype, except that only 2 *lsd1* plants were measured on 12/6/2022. Well-watered (WW) plants were watered on 12/6/2022, 12/9/2022, 12/12/2022, and 12/15/2022. Drought (D) plants were watered on 12/6/2022 and 12/12/2022.

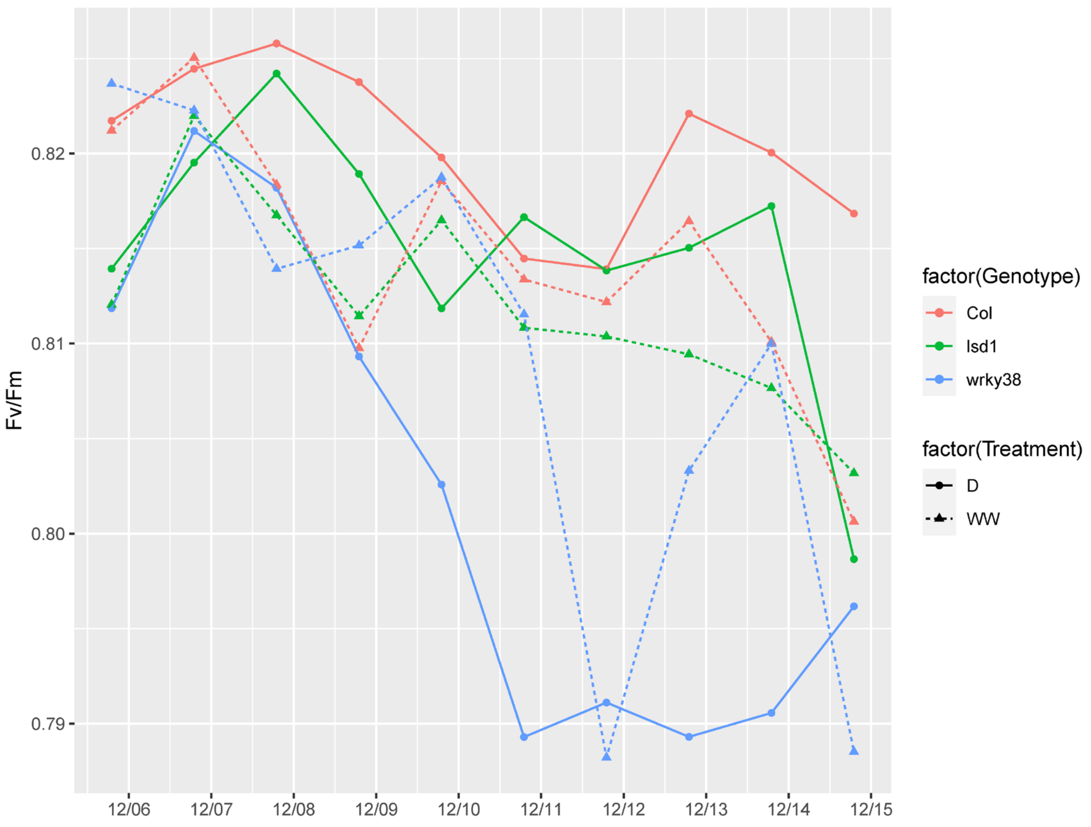

Figure S2 Fv/Fm changes across 10 consecutive days in *Col*, *wrky38*, and *lsd1* measured with LI-COR 600. Measurements were taken at ~0.5h after lights were off. Each point represents mean Fv/Fm from 3-4 plants of the same genotype, except that only 2 *lsd1* plants were measured on 12/6/2022. Well-watered (WW) plants were watered on 12/6/2022, 12/9/2022, 12/12/2022, and 12/15/2022. Drought (D) plants were watered on 12/6/2022 and 12/12/2022. On the watering day, two Fv/Fm measurements were taken right before and ~0.5h after watering.

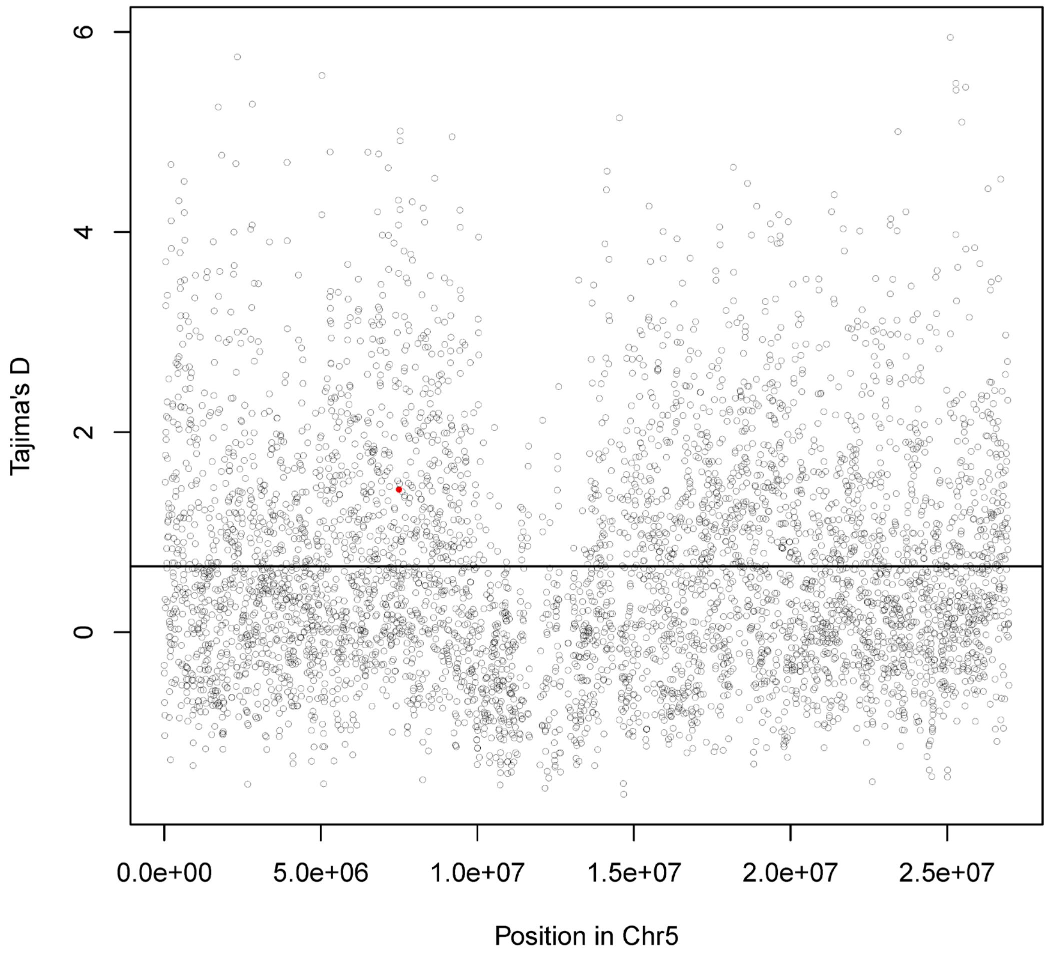

Figure S3 Tajima's D calculated using 5-kb sliding window across chromosome 5. The black line represents the mean Tajima' s D value across chromosome 5. The red dot represents the window that contains *WRKY38*.

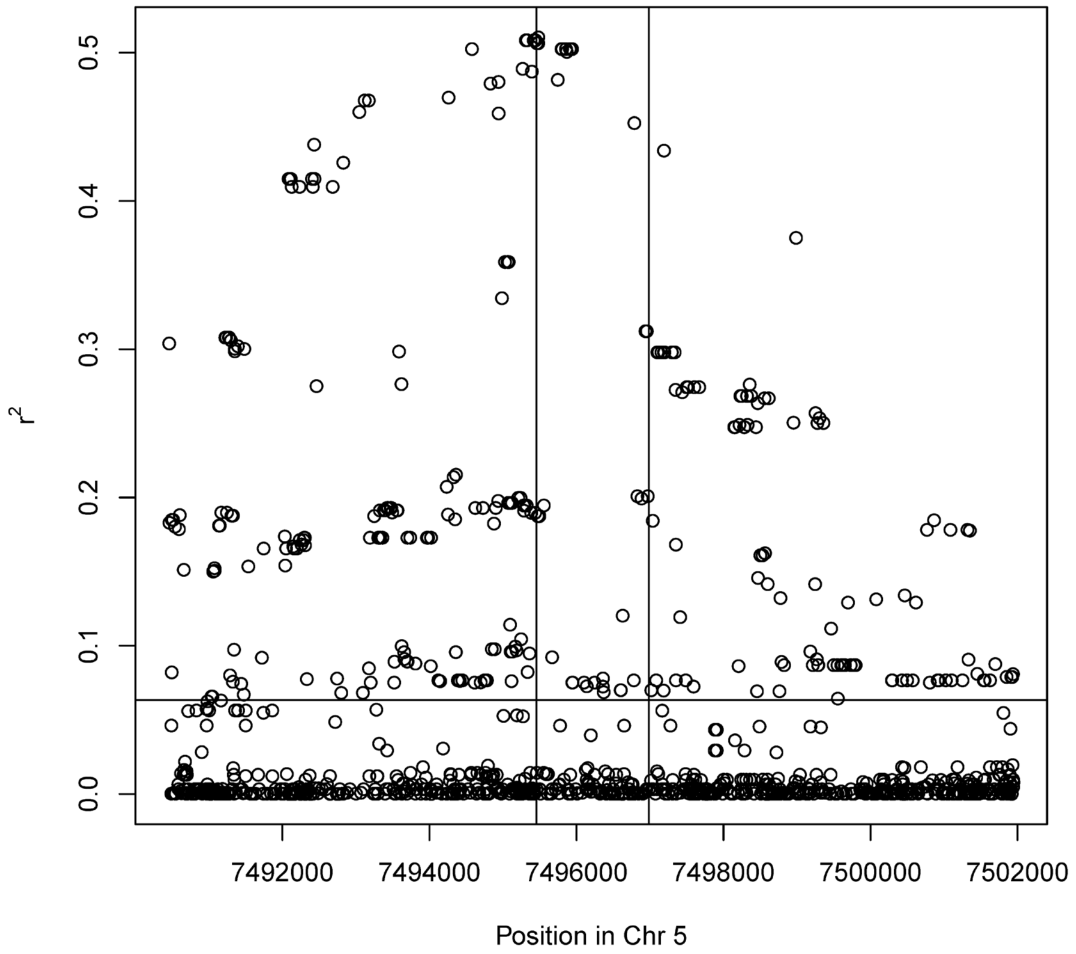

Figure S4 The square of Pearson's correlation coefficient (r^2) between Frameshift$_{7495793}$ and each SNP within 10 kb around *WRKY38*. Vertical lines indicate the boundaries of the *WRKY38* gene, and the horizontal line represents the mean correlation coefficient within the region.

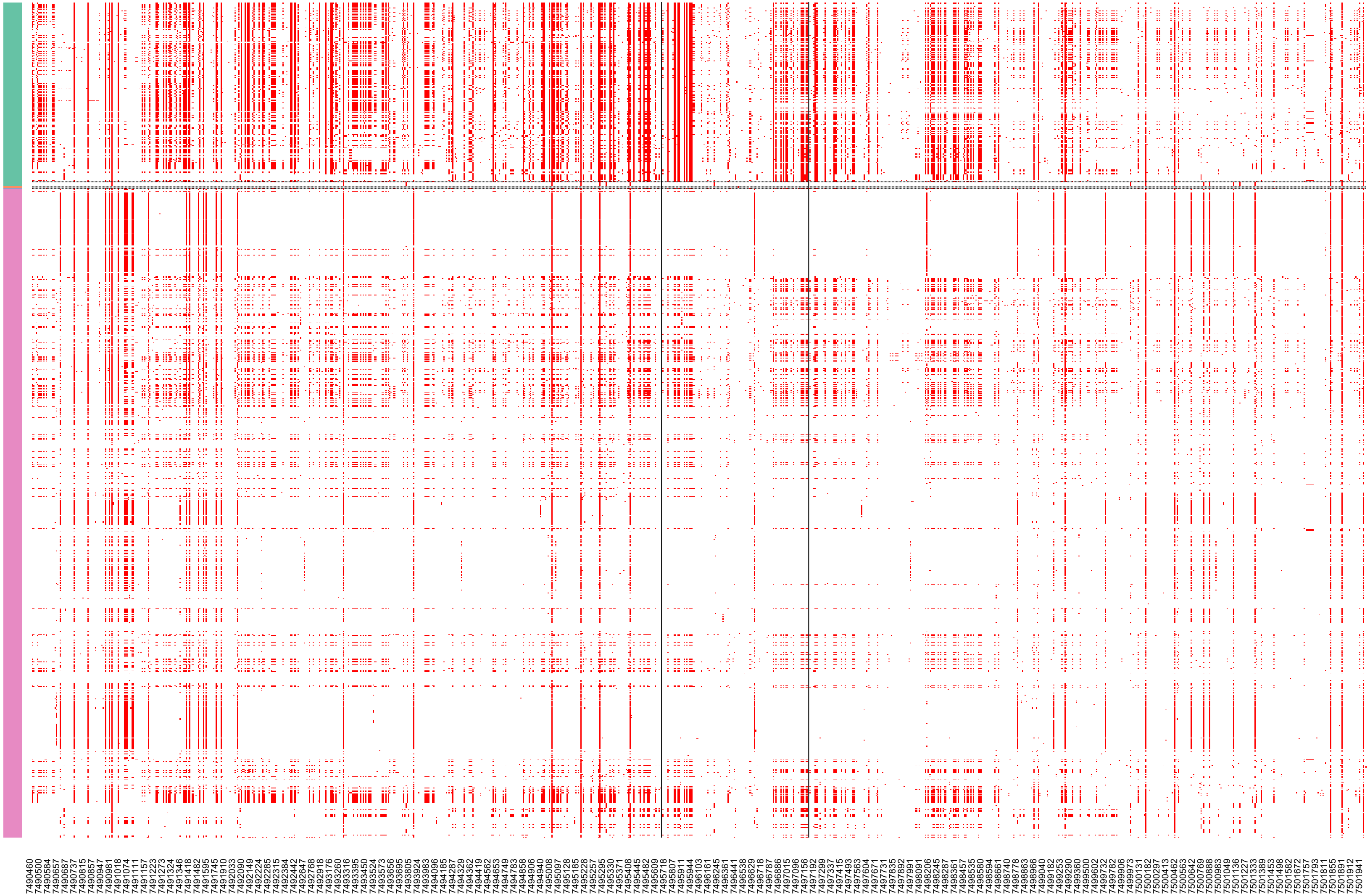

Figure S5 SNP heatmap matrix of 1135 accessions from the 1001 Genomes Project within 10 kb of *WRKY38*. Columns represent SNPs along chromosome 5, rows represent accessions, and white or red squares indicate reference or alternative alleles, respectively. Vertical solid lines mark the boundaries of the *WRKY38* gene. Left strips indicate putative loss-of-function (LoF) variant types for each accession: teal = frameshift, orange = stop gained, purple = stop lost, pink = intact (functional). Horizontal dashed lines separate accessions based on the type or position of the putative LoF variants within the *WRKY38* gene. Accessions are ordered by (1) the type of putative LoF variants, (2) the position of putative LoF variants, and (3) longitude.
